# Physical activity patterns after diagnosis and survival of prognostic colorectal cancer subgroups

**DOI:** 10.1093/jncics/pkaf116

**Published:** 2025-12-15

**Authors:** Karel C Smit, Jeroen W G Derksen, Anne-Sophie van Lanen, Evertine Wesselink, Eric J Th Belt, Maaike Berbée, Marissa Cloos-van Balen, Jan Willem T Dekker, Joyce M van Dodewaard, Joeri Douma, Jan Willem de Groot, Henk K Van Halteren, Mathijs P Hendriks, Ignace H J T De Hingh, Danny Houtsma, Johan J B Janssen, Joop L M Konsten, Maartje Los, Mark P S Sie, Dirkje Sommeijer, Pieter J Tanis, Ankie van der Velden, Liselot Valkenburg-van Iersel, Wouter J Vles, Johannes H W de Wilt, Dieuwertje E Kok, Ellen Kampman, Fränzel J B van Duijnhoven, Miriam Koopman, Anne M May

**Affiliations:** Department of Epidemiology and Health Economics, Julius Center for Health Sciences and Primary Care, University Medical Center Utrecht, Utrecht University, Utrecht, the Netherlands; Department of Epidemiology and Health Economics, Julius Center for Health Sciences and Primary Care, University Medical Center Utrecht, Utrecht University, Utrecht, the Netherlands; Division of Human Nutrition and Health, Wageningen University & Research, Wageningen, the Netherlands; Division of Human Nutrition and Health, Wageningen University & Research, Wageningen, the Netherlands; Department of Surgery, Albert Schweitzer Hospital, Dordrecht, the Netherlands; Department of Radiation Oncology (Maastro), GROW School for Oncology and Reproduction, Maastricht University Medical Centre, Maastricht, the Netherlands; Department of Medical Oncology, Groene Hart Hospital, Gouda, the Netherlands; Department of Surgery, Reinier de Graaf Hospital, Delft, the Netherlands; Department of Medical Oncology, Meander Medical Center, Amersfoort, the Netherlands; Department of Medical Oncology, Medical Center Leeuwarden, Leeuwarden, the Netherlands; Department of Medical Oncology, Isala Hospital, Zwolle, the Netherlands; Department of Medical Oncology, Admiraal de Ruyter Hospital, Goes, the Netherlands; Department of Medical Oncology, Northwest Clinics, Alkmaar, the Netherlands; Department of Surgery, Catharina Hospital, Eindhoven, the Netherlands; Department of Medical Oncology, Haga Hospital, Den Haag, the Netherlands; Department of Medical Oncology, Canisius Wilhelmina Hospital, Nijmegen, the Netherlands; Department of Surgery, Viecuri Hospital, Venlo, the Netherlands; Department of Medical Oncology, St Antonius Hospital, Nieuwegein, the Netherlands; Department of Medical Oncology, ZorgSaam Hospital, Terneuzen, the Netherlands; Department of Internal Medicine, Flevohospital, Almere, the Netherlands; Department of Surgical Oncology and Gastrointestinal Surgery, Erasmus MC, Rotterdam, the Netherlands; Department of Medical Oncology, Tergooi Hospital, Hilversum, the Netherlands; Maastricht University Medical Center, Department of Internal Medicine, Division of Medical Oncology, GROW, Maastricht University, Maastricht, the Netherlands; Department of Surgery, Ikazia Hospital, Rotterdam, the Netherlands; Department of Surgery, Radboud University Medical Center, University of Nijmegen, Nijmegen, the Netherlands; Division of Human Nutrition and Health, Wageningen University & Research, Wageningen, the Netherlands; Division of Human Nutrition and Health, Wageningen University & Research, Wageningen, the Netherlands; Division of Human Nutrition and Health, Wageningen University & Research, Wageningen, the Netherlands; Department of Medical Oncology, University Medical Center Utrecht, Utrecht University, Utrecht, the Netherlands; Department of Epidemiology and Health Economics, Julius Center for Health Sciences and Primary Care, University Medical Center Utrecht, Utrecht University, Utrecht, the Netherlands

## Abstract

**Background:**

Physical activity (PA) is associated with improved overall survival (OS) among colorectal cancer (CRC) patients, but research on PA changes after diagnosis remains limited. This study examines associations between OS and changes in PA from CRC diagnosis onward, across stage- and treatment-related subgroups.

**Methods:**

Data were analyzed from patients in two large CRC cohorts (PLCRC and COLON) enrolled between August 2010 and December 2022 (follow-up until February 1st, 2024). This included 3395 stage I–IIA patients who underwent surgery only, 2406 stage IIB/C–III patients who received (neo-)adjuvant therapy, and 669 metastatic CRC (mCRC) patients. PA was assessed via the validated SQUASH questionnaire at diagnosis (T0), and at 6, 12, and 24 months post-diagnosis (T6 to T24). Moderate-to-vigorous-intensity recreational activity was quantified by calculating Metabolic Equivalent of Task (MET) hours per week. Associations with OS were examined for change (active [tertile 2 and 3] vs inactive [tertile 1]) between timepoints using multivariable Cox proportional hazards models.

**Results:**

Among surgery-only patients, change from inactivity to activity between T0 and T6 was significantly associated with OS (HR = 0.58, 95% CI = 0.35 to 0.96). For (neo-)adjuvantly treated patients, significant associations were observed between T6 and T12 (HR = 0.53, 95% CI = 0.31 to 0.90). Among mCRC patients, a significant association was observed between T6 and T12 (HR = 0.53, 95% CI = 0.29 to 0.99).

**Conclusion:**

Changing from inactivity to activity is significantly associated with prolonged survival during the early months post-diagnosis for surgery-only CRC patients, and later for those undergoing (neo-)adjuvant therapy or with metastatic disease. Validation is warranted in interventional studies.

## Introduction

High levels of physical activity (PA) are associated with a reduced risk of colorectal cancer (CRC).^1^^,^^2^ Observational studies also suggest an association between higher levels of PA and improved survival among CRC patients^3^ and PA interventions after diagnosis of CRC improve quality of life and are safe and feasible.^4^^,^^5^ However, the evidence for improved survival is limited by heterogeneity in the type and timing of PA assessment and included covariates. In addition, only a subset of studies also included subgroup analyses based on stage and/or treatment type.^6-11^ Furthermore, the association between changes in post-diagnostic PA and survival has only been examined in two studies, neither including stage IV patients. The results varied with PA categorization, threshold, and timing of assessment.^10^^,^  ^12^

In 2024, the Global Cancer Update Programme (CUP Global) expert panel graded the current evidence as limited-suggestive for recreational PA and all-cause mortality among all CRC patients, with suggestive evidence for a nonlinear inverse association.^3^ They recommended randomized controlled trials and observational studies with standardized repeated measures in CRC subgroups.

Recently, the first randomized controlled trial of a 3-year structured exercise program starting 2–6 months after adjuvant chemotherapy in colon cancer patients showed significantly longer disease-free survival,^13^ marking an important advance for this subgroup. Broader evidence across the wider CRC population, however, remains needed.

Therefore, the main aim of our study was to investigate the association of PA changes with survival in distinct CRC prognostic groups: those with non-metastatic disease indicated for surgery only, those receiving additional (neo-)adjuvant treatment, and those with stage IV CRC regardless of treatment.

## Methods

### Study population

We combined data from 2 large cohorts: the Prospective Dutch Colorectal Cancer (PLCRC) cohort (NCT02070146, ClinicalTrials.gov) and the COlorectal cancer Longitudinal Observational study on Nutritional and lifestyle factors that may influence colorectal tumor recurrence, survival and quality of life—COLON cohort (NCT03191110, ClinicalTrials.gov). PLCRC was approved by the Medical Research Ethics Committee of Utrecht, the Netherlands (METC 12-510), and COLON was approved by the Committee on Research involving Human Subjects, region Arnhem-Nijmegen, the Netherlands (2009–349). All patients provided informed consent. Cohort designs have been described elsewhere.^14^^,^^15^ We included patients enrolled between February 2016 and December 2022 (PLCRC) and between August 2010 and February 2020 (COLON). Inclusion/exclusion criteria and further study characteristics can be found in Table S1. Briefly, adults (≥18 years) with any CRC were recruited and consented to repeated questionnaires at standardized intervals. Patients were excluded from this analysis when they did not complete their first questionnaire within 60 days of primary CRC diagnosis. The final population comprised 5782 PLCRC and 1731 COLON patients.

### Physical activity assessment

PA was measured with the validated SQUASH questionnaire^16^ at diagnosis and 6, 12, and 24 months after diagnosis (T0-T24). Patients reported habitual PA during a typical week in the months preceding the questionnaire. At T12, in the COLON cohort, only patients who were receiving adjuvant chemotherapy were surveyed.

### Covariates assessment

Clinical data were obtained from the Netherlands Cancer Registry (NCR), extracted by trained data managers from electronic health records and collected for (treatment of) the primary CRC diagnosis (ie, first disease episode). Information on comorbidity, treatment changes, and possible recurrence and/or progression of disease was largely unavailable and therefore not used in current analyses. Questionnaire data provided body mass index (BMI) and stoma presence, assessed concurrently with the SQUASH.

### Study endpoint

The primary endpoint was overall survival (OS), defined as time from CRC diagnosis to death from any cause. Vital status was obtained through annual linkage with the municipal population registry, last updated on February 1^st^ 2024, which served as the censoring date.

### Data analysis

To reduce heterogeneity, we created three subgroups in which analyses were performed separately:

1. Stage I-IIA CRC patients who received surgical resection (henceforth “Surgery-only CRC”)
2. Stage IIB/C & stage III colon cancer patients treated with surgery plus adjuvant therapy, and stage IIB/C & stage III rectal cancer patients receiving neo-adjuvant therapy (henceforth: “(Neo-)adjuvantly treated CRC”)
3. All stage IV CRC patients regardless of treatment (henceforth: “mCRC”)

Cox proportional hazards (PH) regression models estimated hazard ratios (HRs) for PA variables. Models adjusted for age, sex (male, female), primary tumor site (colon, rectum), cohort (PLCRC, COLON), BMI (18.5-25 kg/m^2^, other; time-varying), and stoma (no, yes; time-varying). For mCRC analyses, additional covariates were number of metastases (1, >1), liver-only metastasis (yes, no), surgery of primary tumor (yes, no), metastasectomy (yes, no), and additional treatment before first progression (none, chemotherapy, radiotherapy, both). Analyses were conducted for each separate timepoint, and for changes in PA between subsequent timepoints. Timepoint results are reported for comparison with previous studies, but the main focus is PA change.

Completed questionnaires were quantified by assigning Metabolic Equivalent of Task (MET, 1 MET is the rate of energy used while at rest) values to each activity, using the Ainsworth compendium.^17^ Analyses focused on moderate-to-vigorous-intensity recreational PA (MET ≥3). PA was assessed continuously and categorically. Details on the categorization and calculation of PA variables have been previously reported.^18^

PA categories were based on tertiles in a representative Dutch population sample (Statistics Netherlands [CBS] health surveys, 2016-2019).^19-22^ This sample was matched 1:1 with the CRC cohort by sex and a 10-year age range. We used this method to set representative population-based values for PA tertiles. Consequently, the categorization within our study population is not evenly distributed, and we refer to the three groups as low, moderate, and high recreational PA. In timepoint analyses, both moderate and high PA were compared with low PA. For PA change analyses, four categories were created, based on PA status across 2 subsquent timepoints: (1) *Remains inactive* (low to low), (2) *Becomes inactive* (moderate/high to low), (3) *Becomes active* (low to moderate/high), (4) *Remains active* (moderate/high to moderate/high). “Remains inactive” was used as the reference category.

Linearity of continuous PA variables with OS (both timepoint and change-between-timepoint analyses) was evaluated using restricted cubic splines (RCS) with knots placed at the 10^th^, 50^th^ and 90^th^ percentiles, and truncation at the 1st and 99th percentile.

Analyses were not performed when the number of events was less than the total number of variables divided by 10, resulting in exclusion of change analyses between T12 and T24 for surgery-only and mCRC patients. The PH assumption was tested both visually and by using scaled Schoenfeld residuals.

### Supplemental analyses

To enhance comparability with prior studies, we repeated Cox and spline models for additional PA metrics: total PA (MET-hours/week), total moderate-to-vigorous-intensity PA (MVPA, MET-hours/week), and adherence to the American College of Sports Medicine (ACSM) PA guidelines.^23^ Associations for recreational PA with OS were repeated for all stage I-III and all stage I-IV CRC patients combined.

### Sensitivity analyses

Sensitivity analyses included: (1) excluding patients who died or were lost to follow-up within 6 months of their last questionnaire; (2) adjusting for additional variables: smoking (current, former, never), education (low, moderate, high), and marital status (single, married/in-law), omitted from main models due to limited events at later timepoints to preserve model stability and comparability across CRC subgroups; and (3) adjusting for functional status using the revised physical functioning (PF2) subscale of the validated European Organization for the Research and Treatment of Cancer Quality of Life Questionnaire-Core 30 (EORTC QLQ-C30, version 3.0)^24^ at the time of (first) PA assessment. The PF2 subscale captures patients’ ability to perform strenuous and moderate activities, as well as their capacity to walk and carry out daily tasks. Scores (0-100) were classified as good (>66.7) or poor (≤66.7). Because functional status and PA are conceptually closely related indicators of patient well-being and capacity,^25^ this was considered only as a sensitivity analysis. Univariate PA results are also shown for comparison.

Missing data were not imputed under the Missing Not At Random (MNAR) assumption, given limited information on disease and treatment follow-up. Differences between patients returning 1 and ≥3 questionnaires were assessed from baseline characteristics. To assess the range of uncertainty due to missing data, patients with missing questionnaire data but >3 months follow-up after their last questionnaire were reassigned to both low and moderate/high activity. PA change analyses were repeated with these classifications.

Analyses were performed using R (version 4.4.0).^26^ Splines were generated with the RMS package,^27^ and figures with the ggplot2^28^ package. All statistical tests were performed 2-sided with an alpha level of <.05.

## Results

Among surgery-only (*n* = 3395), (neo-)adjuvantly treated (*n* = 2406), and mCRC patients (*n* = 669), median age at diagnosis was 68 (IQR = 61-74), 64 (56-70), and 64 (55-71) years, and 60%, 61% and 64% were male, respectively (Table 1). Deaths totaled 280 (8%), 378 (16%) and 446 (67%) during median follow-up of 43 (IQR = 27-70), 46 (27-71), and 24 (14-39) months. At diagnosis, median recreational PA in CRC subgroups was comparable to the general population (27, 25, 24 vs 25), whereas median total PA was lower (90, 99, 90 vs 107). Population-based tertiles yielded similar recreational PA distributions in CRC patients, but more patients classified as inactive for total PA, particularly at T6 for higher-stage CRC: 54% for (neo-)adjuvantly treated, and 67% for stage IV CRC (Figure S1). At T24, PA data were missing for 43%, 38%, and 37% of CRC patients due to reduced response or not yet reaching that timepoint.

**Table 1.** Characteristics of colorectal cancer patients at diagnosis and the general Dutch population, matched on sex and a 10-year age range.

	Surgery only CRC		(Neo-)adjuvantly treated CRC		mCRC		General population
	Low PA	Moderate and high PA	Low PA	Moderate and high PA	Low PA	Moderate and High PA	Total
	(*n* = 986)	(*n* = 2346)	(*n* = 716)	(*n* = 1659)	(*n* = 238)	(*n* = 424)	(*n* = 7513)
Sex							
Male	581 (59%)	1427 (61%)	443 (62%)	1001 (60%)	150 (63%)	275 (65%)	4608 (61%)
Female	405 (41%)	919 (39%)	273 (38%)	658 (40%)	88 (37%)	149 (35%)	2905 (39%)
Age							
Median (IQR)	68 (60-75)	68 (62-73)	63 (55-71)	64 (57-70)	64 (54-74)	64 (56-70)	66 (59-73)
Recreational PA (MET-hrs/wk)^±^							
Median (IQR)	4.5 (0.0-9.0)	40 (25-61)	5.8 (0.0-9.9)	37 (24-58)	4.0 (0.0-9.0)	36 (25-55)	25 (9.0-51)
Total PA (MET-hrs/wk)^±^							
Median (IQR)	56 (19-96)	104 (66-148)	71 (35-107)	110 (74-156)	58 (20-107)	105 (69-148)	107 (63-162)
Total MVPA (MET-hrs/wk)^±^							
Median (IQR)	16 (6-42)	62 (39-101)	22 (9-52)	63 (39-103)	17 (6-53)	60 (38-97)	52 (22-98)
PA guideline^±^							
Non adherence	834 (85%)	1119 (48%)	600 (84%)	756 (46%)	206 (87%)	207 (49%)	4288 (57%)
Adherence	152 (15%)	1227 (52%)	116 (16%)	903 (54%)	32 (13%)	217 (51%)	3225 (43%)
BMI^±^							
Median (IQR)	27 (24-30)	26 (24-29)	27 (24,-30)	26 (23-28)	26 (23-29)	25 (23-28)	26 (24-29)
Healthy	319 (33%)	950 (41%)	247 (35%)	723 (44%)	94 (40%)	212 (50%)	2920 (39%)
Unhealthy	662 (67%)	1383 (59%)	465 (65%)	934 (56%)	142 (60%)	210 (50%)	4593 (61%)
Smoking status^±^							
Never	328 (33%)	782 (33%)	232 (33%)	609 (37%)	79 (33%)	161 (38%)	2488 (33%)
Prior	548 (56%)	1437 (61%)	396 (56%)	951 (57%)	124 (52%)	242 (57%)	3722 (50%)
Current	108 (11%)	123 (5%)	85 (12%)	97 (6%)	34 (14%)	21 (5%)	1303 (17%
Education^±^							
Low	425 (43%)	733 (31%)	249 (35%)	474 (29%)	92 (39%)	111 (26%)	2686 (37%)
Intermediate	291 (30%)	724 (31%)	248 (35%)	486 (29%)	67 (28%)	122 (29%)	2691 (37%)
High	263 (27%)	881 (38%)	211 (30%)	695 (42%)	77 (33%)	189 (45%)	1962 (27%)
Marital status							
Single	210 (21%)	434 (18%)	126 (18%)	274 (17%)	50 (21%)	77 (18%)	1819 (25%)
Married/common-law	776 (79%)	1912 (82%)	589 (82%)	1384 (83%)	187 (79%)	346 (82%)	5554 (75%)
Primary tumor location							–
Colon	812 (82%)	1890 (81%)	426 (59%)	1057 (64%)	167 (70%)	279 (66%)	–
Rectum	174 (18%)	456 (19%)	290 (41%)	602 (36%)	71 (30%)	145 (34%)	–
Surgery primary tumor*							–
No	0 (0%)	0 (0%)	49 (7%)	92 (6%)	116 (49%)	166 (39%)	–
Yes	986 (100%)	2346 (100%)	667 (93%)	1567 (94%)	122 (51%)	258 (61%)	–
Stoma directly after surgery							–
No	869 (89%)	2076 (90%)	480 (68%)	1188 (72%)	163 (71%)	285 (71%)	–
Yes	111 (11%)	240 (10%)	230 (32%)	452 (28%)	65 (29%)	117 (29%)	–
Additional treatment*							–
None	986 (100%)	2346 (100%)	0 (0%)	0 (0%)	52 (22%)	90 (21%)	–
Radiotherapy	0 (0%)	0 (0%)	81 (11%)	206 (12%)	6 (3%)	24 (6%)	–
Chemotherapy	0 (0%)	0 (0%)	446 (62%)	1105 (67%)	141 (59%)	223 (53%)	–
Chemo- and radiotherapy	0 (0%)	0 (0%)	189 (26%)	348 (21%)	39 (16%)	87 (21%)	–
Number of metastases^§^							–
1	–	–	–	–	147 (62%)	306 (72%)	–
>1	–	–	–	–	90 (38%)	117 (28%)	–
Liver-only metastasis^§^							–
Yes	–	–	–	–	98 (41%)	233 (55%)	–
No	–	–	–	–	139 (59%)	190 (45%)	–
Metastasectomy*							–
No	–	–	–	–	149 (63%)	224 (53%)	–
Yes	–	–	–	–	89 (37%)	200 (47%)	–

Percentages may not equal 100 due to rounding. PA categorization was based on cut-offs from tertiles created in an age- and sex-matched sample of the general population. Low: tertile 1, moderate: tertile 2, high: tertile 3. For this table, PA measurement at diagnosis (T0) was used. Healthy BMI: 18.5-25, unhealthy BMI: <18.5, >25. Low education: elementary and lower vocational education, intermediate: secondary education, high: higher vocational and university education.

* Treatment information is based on treatment for the primary CRC diagnosis (ie, first disease episode). Information on changes of treatment, and possible recurrence and/or progression of disease was not available.

± Percentages of missingness for surgery only CRC, (neo-)adjuvant CRC and mCRC is 1.9%, 1.3% and 1.0% for physical activity variables; 0.5%, 0.3%, and 0.6% for BMI; 0.2%, 0.2%, and 0.1% for smoking status; 0.5%, 0.5%, and 0.6% for education; 0%, 0.1%, and 0.3% for marital status; and 1.1%, 1.0% and 4.8% for stoma.

§ Location and number of metastases was missing for 0.3% of mCRC patients.

Abbreviations: CRC = colorectal cancer; mCRC = metastatic colorectal cancer; IQR = interquartile range; PA = physical activity; MET-hrs/wk = metabolic equivalent of task-hours per week; BMI = body mass index.

### Association between recreational physical activity and survival

**Figure 1.**
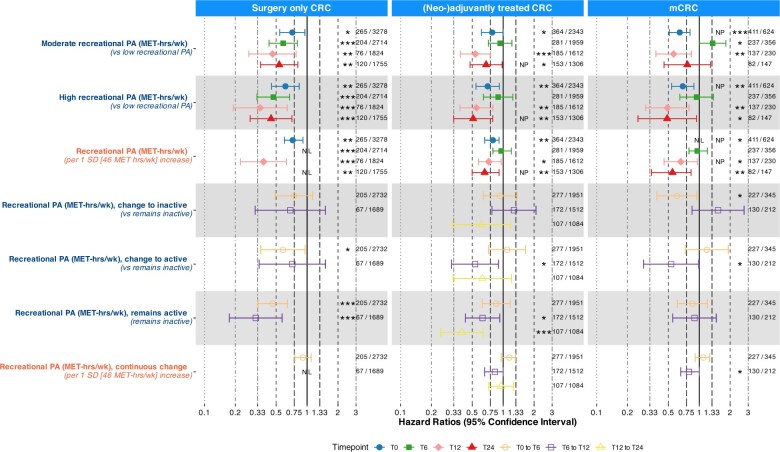
Associations of recreational physical activity (MET-hours/week) with overall survival from Cox proportional hazard models. PA categorization was based on cut-offs from tertiles created in an age- and sex-matched sample of the general population. Low: tertile 1, moderate: tertile 2, high: tertile 3. Remains inactive: low at both timepoints; change to inactive: moderate/high at first- and low at second timepoint; change to active: low at first- and moderate/high at second timepoint; remains active: moderate/high at both timepoints. Standard deviations were also calculated based on the general population sample. T12 to T24 change analyses were removed for surgery only CRC and stage IV CRC due to limited events. Numbers depict deaths/total patients in analysis. Stars indicate significant associations (*: *P* = .01 to <.05; **: *P* = .001 to <.01; *** *P* <.001). NL indicates a nonlinear association, NP that the proportional hazards assumption is violated. Fixed covariates: age (continuous), sex (male, female), primary tumor site (colon, rectum), cohort (PLCRC, COLON). Covariates at time of PA measurement: BMI (18.5-25, other), stoma (yes, no). Additional covariates in stage IV analyses: number of metastases (1, >1), liver-only metastasis (yes, no), surgery of primary tumor (yes, no), metastasectomy (yes, no), additional treatment during first disease episode (none, chemotherapy, radiotherapy, both). Abbreviations: CRC = colorectal cancer; PA = physical activity; HR = hazard ratio; CI = confidence interval; MET-hrs/wk = metabolic equivalent of task-hours per week; SD = standard deviation; T0 = diagnosis; T6 = 6 months after diagnosis; T12 = 12 months after diagnosis; T24 = 24 months ater diagnosis.

**Figure 2.**
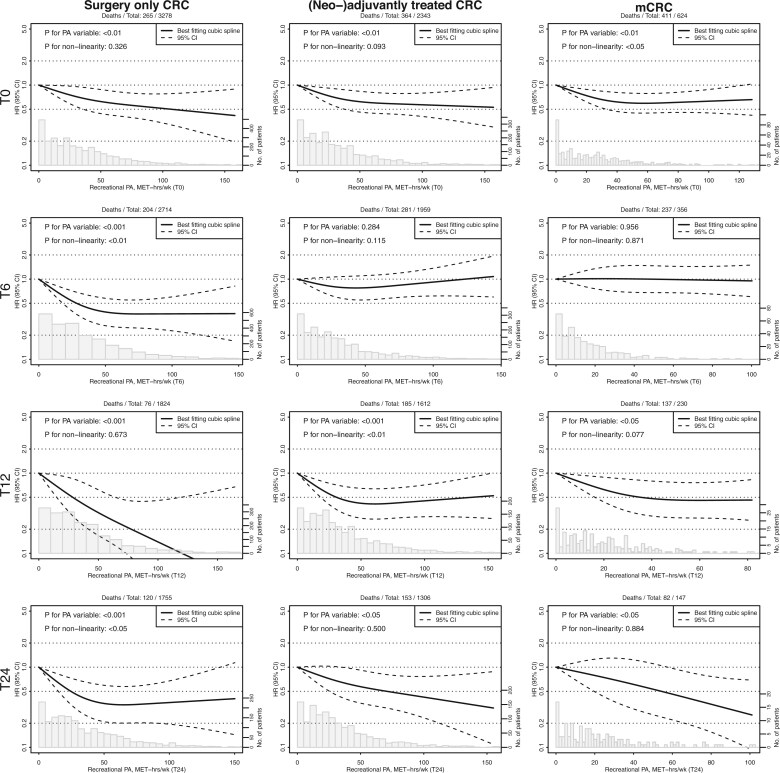
Restricted cubic spline analyses of Cox proportional hazard models for timepoint recreational physical activity (MET-hours/week) and overall survival. Plot curves were estimated using restricted cubic spline regressions with three knots placed at fixed percentiles (10%, 50%, and 90%) of the physical activity variable, and 0 MET-hours was chosen as the reference category. Spline plots were truncated at the 1^st^ and 99^th^ percentile. Note that the number of deaths is below the number of variables divided by 10 for T24 for stage IV CRC analyses, possibly resulting in unstable estimates. Abbreviations: CRC = colorectal cancer; PA = physical activity; HR = hazard ratio; CI = confidence interval; MET-hrs/wk = metabolic equivalent of task-hours per week; T0 = diagnosis; T6 = 6 months after diagnosis; T12 = 12 months after diagnosis; T24 = 24 months after diagnosis.

**Figure 3.**
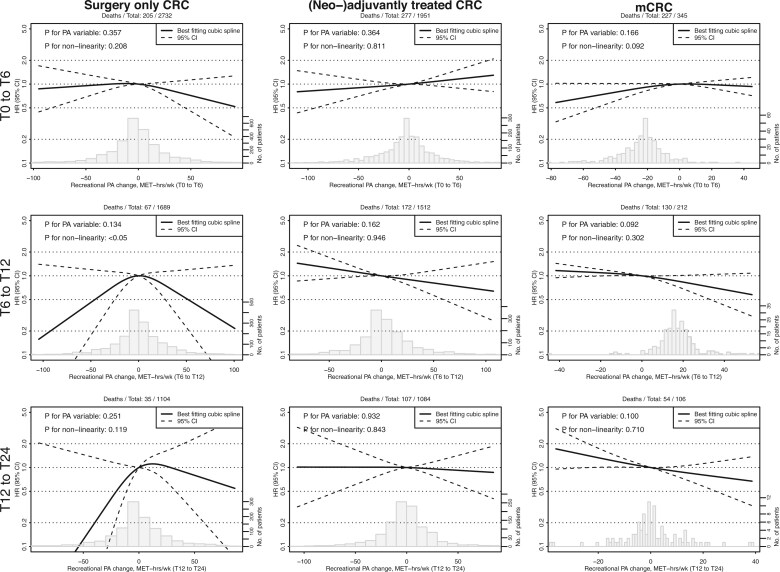
Restricted cubic spline analyses of Cox proportional hazard models for changes between subsequent timepoints in recreational physical activity (MET-hours/week) and overall survival. Plot curves were estimated using restricted cubic spline regressions with three knots placed at fixed percentiles (10%, 50%, and 90%) of the physical activity variable, and 0 MET-hours was chosen as the reference category. Spline plots were truncated at the 1^st^ and 99^th^ percentile. Note that the number of deaths is below the number of variables divided by 10 for T12 and T24 for both surgery only CRC and mCRC analyses, possibly resulting in unstable estimates. Abbreviations: CRC = colorectal cancer; PA = physical activity; HR = hazard ratio; CI = confidence interval; MET-hrs/wk = metabolic equivalent of task-hours per week; T0 = diagnosis; T6 = 6 months after diagnosis; T12 = 12 months after diagnosis; T24 = 24 months after diagnosis.

In surgery-only CRC patients, both moderate and high vs low PA were consistently associated with improved OS at all timepoints (Figure 1, Table 2). Compared to remaining inactive, changing PA from inactive to active was associated with prolonged survival between T0 and T6 (HR = 0.58, 95% CI = 0.35 to 0.96), as was remaining active between both T0 and T6 (HR = 0.46, 95% CI = 0.33 to 0.64), and T6 and T12 (HR = 0.32, 95% CI = 0.18 to 0.57). Continuous PA was significantly associated with improved OS at all timepoints, with nonlinear patterns at T6 and T24, indicating limited benefit above ∼50 MET-hrs/wk (Figure 2, Table 3). No significant associations were observed for continuous PA change between subsequent timepoints, and a nonlinear pattern was observed between T6 and T12 (Figure 3, Table 3).

**Table 2.** Associations from Cox proportional hazard models for associations of (change of) recreational physical activity (MET-hours per week) with overall survival.

		Surgery only CRC			(Neo-)adjuvantly treated CRC			mCRC		
Time	Category	FU (IQR)	Death/total	HR (95% CI)	FU (IQR)	Death/total	HR (95% CI)	FU (IQR)	Death/total	HR (95% CI)
*T0*	*Low*	48 (28-72)	109/975	*Ref.*	48 (30-75)	139/706	*Ref.*	19 (11-32)	163/225	*Ref.*
T0	Moderate	44 (28-72)	90/1186	**0.718 (0.542 to 0.952)**	46 (27-70)	128/899	**0.782 (0.614 to 0.996)**	28 (17-43)	138/222	**0.645 (0.511 to 0.813)**
T0	High	40 (25-65)	66/1117	**0.615 (0.451 to 0.838)**	44 (28-68)	97/739	**0.706 (0.543 to 0.917)**	26 (15-42)	110/177	**0.691 (0.538 to 0.888)**
T0	Continuous	43 (27-69)	265/3278	**0.724 (0.601 to 0.912)**	46 (28-71)	364/2344	**0.794 (0.660 to 0.912)**	24 (14-40)	411/624	**Non-linear, significant**
*T6*	*Low*	*52 (30-81)*	*96/775*	*Ref.*	*57 (33-85)*	*112/647*	*Ref.*	*34 (21-57)*	*95/146*	*Ref.*
T6	Moderate	48 (28-74)	65/1050	**0.585 (0.426 to 0.804)**	51 (31-78)	112/799	0.934 (0.717 to 1.216)	31 (19-47)	100/142	**1.351 (1.006 to 1.816)**
T6	High	41 (27-67)	43/889	**0.469 (0.325 to 0.676)**	41 (28-62)	57/514	0.893 (0.642 to 1.242)	34 (24-46)	42/68	0.941 (0.647 to 1.370)
T6	Continuous	47 (28-74)	204/2714	**Non-linear, significant**	50 (30-76)	281/1960	0.955 (0.794 to 1.202)	33 (21-49)	237/356	0.955 (0.794 to 1.202)
*T12*	*Low*	38 (26-55)	37/455	*Ref.*	48 (31-70)	72/405	*Ref.*	31 (22-44)	58/79	*Ref.*
T12	Moderate	37 (24-53)	23/699	**0.461 (0.271 to 0.782)**	50 (29-74)	64/670	**0.536 (0.382 to 0.753)**	39 (30-56)	53/92	**0.563 (0.378 to 0.840)**
T12	High	35 (24-53)	16/670	**0.350 (0.191 to 0.640)**	47 (29-65)	49/538	**0.549 (0.379 to 0.794)**	39 (31-56)	26/59	**0.489 (0.301 to 0.794)**
T12	Continuous	37 (24-54)	76/1824	**0.377 (0.224 to 0.630)**	48 (29-69)	185/1613	**Non-linear, significant**	36 (26-52)	137/230	**0.660 (0.454 to 0.955)**
*T24*	*Low*	*62 (40-95)*	*55/477*	*Ref.*	*63 (40-89)*	*63/350*	*Ref.*	*46 (34-67)*	*34/50*	*Ref.*
T24	Moderate	60 (40-90)	38/662	**0.535 (0.352 to 0.813)**	60 (40-85)	59/549	**0.682 (0.476 to 0.978)**	46 (36-65)	33/57	0.765 (0.454 to 1.288)
T24	High	55 (37-83)	27/616	**0.446 (0.279 to 0.712)**	57 (40-79)	31/408	**0.510 (0.329 to 0.791)**	53 (42-68)	15/40	**0.487 (0.253 to 0.939)**
T24	Continuous	59 (39-88)	120/1755	**Non-linear, significant**	60 (40-84)	153/1307	**0.660 (0.499 to 0.912)**	48 (36-67)	82/147	**0.548 (0.343 to 0.832)**
*T0 to T6*	*Remains inactive*	50 (29-79)	63/477	*Ref.*	57 (33-86)	59/341	*Ref.*	29 (18-41)	53/77	*Ref.*
T0 to T6	Change to inactive	51 (30-76)	34/312	0.746 (0.491 to 1.134)	56 (33-83)	51/311	0.933 (0.640 to 1.358)	42 (25-65)	39/66	**0.605 (0.386 to 0.948)**
T0 to T6	Change to active	49 (30-74)	21/299	**0.582 (0.354 to 0.956)**	48 (33-73)	36/222	1.092 (0.720 to 1.656)	27 (17-47)	26/35	1.183 (0.730 to 1.917)
T0 to T6	Remains active	42 (26-70)	87/1644	**0.462 (0.332 to 0.643)**	46 (28-68)	131/1078	0.855 (0.625 to 1.170)	34 (22-47)	109/167	0.855 (0.610 to 1.200)
T0 to T6	Continuous change	45 (28-72)	205/2732	0.912 (0.758 to 1.096)	50 (30-75)	277/1952	1.148 (0.955 to 1.317)	33 (21-49)	227/345	1.096 (0.912 to 1.258)
*T6 to T12*	*Remains inactive*	*37 (26-55)*	*21/255*	*Ref.*	*48 (33-74)*	*39/233*	*Ref.*	*32 (23-44)*	*31/43*	*Ref.*
T6 to T12	Change to inactive	39 (25-53)	9/161	0.686 (0.312 to 1.510)	48 (28-68)	27/144	1.274 (0.777 to 2.088)	31 (20-45)	24/32	1.524 (0.850 to 2.732)
T6 to T12	Change to active	40 (26-54)	11/177	0.718 (0.341 to 1.511)	59 (35-83)	22/227	**0.532 (0.314 to 0.900)**	44 (35-66)	19/43	**0.532 (0.287 to 0.987)**
T6 to T12	Remains active	35 (24-52)	26/1096	**0.316 (0.175 to 0.571)**	46 (29-68)	84/909	**0.630 (0.429 to 0.927)**	36 (26-51)	56/94	0.899 (0.552 to 1.465)
T6 to T12	Continuous change	37 (24-53)	67/1689	Non-linear, non-significant	49 (30-71)	172/1513	0.832 (0.660 to 1.000)	36 (27-52)	130/212	**0.794 (0.660 to 1.000)**
*T12 to T24*	*Remains inactive*	*48 (35-60)*	*13/155*	*Ref.*	*58 (39-78)*	*26/137*	*Ref.*	*47 (35-64)*	*14/20*	*Ref.*
T12 to T24	Change to inactive	41 (33-59)	3/112	NA	56 (38-81)	14/121	0.616 (0.320 to 1.186)	48 (42-62)	7/13	NA
T12 to T24	Change to active	44 (35-58)	4/112	NA	55 (39-76)	14/117	0.626 (0.326 to 1.205)	53 (36-79)	5/7	NA
T12 to T24	Remains active	43 (34-59)	15/725	NA	57 (38-77)	53/710	**0.395 (0.246 to 0.636)**	49 (38-63)	28/66	NA
T12 to T24	Continuous change	44 (34-59)	35/1104	NA	57 (38-78)	107/1085	0.955 (0.724 to 1.258)	48 (36-63)	54/106	NA

Effect estimates for continuous activity are shown per 1 SD increase (46 MET-hrs/wk).

PA categorization was based on cut-offs from tertiles created in an age- and sex-matched sample of the general population. Low (reference group): tertile 1, moderate: tertile 2, high: tertile 3. Remains inactive (reference group): low at both timepoints; change to inactive: moderate/high at first- and low at second timepoint; change to active: low at first- and moderate/high at second timepoint; remains active: moderate/high at both timepoints.

Bold values illustrate statistically significant associations.

Abbreviations: CRC = colorectal cancer; FU = follow-up time in months; IQR = interquartile range; HR = hazard ratio; CI = confidence interval; PA = physical activity; MET-hrs/wk = metabolic equivalent of task-hours per week.

**Table 3.** Restricted cubic spline results from Cox proportional hazard models of recreational physical activity (MET-hours per week) with overall survival.

		Surgery only CRC	(Neo-)adjuvantly treated CRC	mCRC
Timepoint	MET-hrs/wk	HR (95% CI)	HR (95% CI)	HR (95% CI)
T0	0	Ref.	Ref.	Ref.
	**3**	**0.938 (0.883 to 0.996)**	**0.928 (0.878 to 0.981)**	**0.949 (0.920 to 0.979)**
	**9**	**0.903 (0.821 to 0.994)**	**0.894 (0.823 to 0.972)**	**0.866 (0.794 to 0.944)**
	**25**	**0.765 (0.602 to 0.973)**	**0.733 (0.587 to 0.916)**	**0.704 (0.572 to 0.865)**
	**50**	**0.622 (0.440 to 0.879)**	**0.629 (0.468 to 0.846)**	**0.597 (0.450 to 0.792)**
	**70**	**0.580 (0.410 to 0.820)**	**0.586 (0.435 to 0.788)**	**0.599 (0.452 to 0.794)**
T6	0	Ref.	Ref.	Ref.
	3	**0.972 (0.959 to 0.985)**	1.165 (0.958 to 1.416)	1.002 (0.954 to 1.053)
	9	**0.779 (0.691 to 0.877)**	1.102 (0.972 to 1.248)	1.006 (0.877 to 1.155)
	25	**0.550 (0.417 to 0.726)**	0.979 (0.950 to 1.008)	1.012 (0.739 to 1.385)
	50	**0.391 (0.264 to 0.579)**	0.944 (0.839 to 1.063)	0.999 (0.679 to 1.470)
	70	**0.370 (0.249 to 0.550)**	1.005 (0.828 to 1.219)	0.973 (0.658 to 1.439)
T12	0	Ref.	Ref.	Ref.
	3	**0.929 (0.871 to 0.991)**	**0.888 (0.835 to 0.944)**	**0.874 (0.787 to 0.970)**
	9	**0.822 (0.693 to 0.975)**	**0.808 (0.724 to 0.903)**	**0.797 (0.670 to 0.949)**
	25	**0.525 (0.316 to 0.872)**	**0.566 (0.423 to 0.759)**	**0.558 (0.363 to 0.858)**
	50	**0.312 (0.161 to 0.603)**	**0.423 (0.276 to 0.648)**	**0.460 (0.277 to 0.763)**
	70	**0.234 (0.114 to 0.480)**	**0.414 (0.268 to 0.640)**	**0.458 (0.275 to 0.762)**
T24	0	Ref.	Ref.	Ref.
	3	**0.905 (0.855 to 0.957)**	0.955 (0.906 to 1.008)	0.948 (0.846 to 1.062)
	9	**0.761 (0.653 to 0.886)**	0.863 (0.728 to 1.023)	0.898 (0.716 to 1.126)
	25	**0.510 (0.353 to 0.737)**	0.708 (0.489 to 1.026)	0.733 (0.415 to 1.294)
	50	**0.350 (0.207 to 0.591)**	**0.559 (0.347 to 0.899)**	0.541 (0.260 to 1.124)
	70	**0.339 (0.201 to 0.574)**	**0.489 (0.300 to 0.795)**	**0.452 (0.215 to 0.948)**
T0 to T6	−50	0.955 (0.693 to 1.317)	0.883 (0.653 to 1.194)	0.702 (0.484 to 1.019)
	−25	1.004 (0.876 to 1.150)	0.949 (0.867 to 1.038)	0.892 (0.784 to 1.016)
	−9	1.016 (0.966 to 1.068)	0.977 (0.945 to 1.010)	0.968 (0.923 to 1.016)
	0	Ref.	Ref.	Ref.
	9	0.953 (0.888 to 1.023)	1.026 (0.988 to 1.065)	1.002 (0.967 to 1.038)
	25	0.891 (0.757 to 1.047)	1.085 (0.945 to 1.246)	0.977 (0.862 to 1.107)
	50	0.730 (0.474 to 1.123)	1.136 (0.906 to 1.426)	0.946 (0.761 to 1.177)
T6 to T12	-50	0.480 (0.193 to 1.193)	1.210 (0.937 to 1.563)	1.154 (0.954 to 1.398)
	-25	0.765 (0.523 to 1.120)	1.126 (0.969 to 1.309)	1.110 (0.986 to 1.251)
	-9	0.953 (0.853 to 1.064)	1.038 (0.997 to 1.080)	1.056 (1.005 to 1.111)
	0	Ref.	Ref.	Ref.
	9	0.970 (0.876 to 1.074)	0.963 (0.925 to 1.004)	0.922 (0.850 to 1.000)
	25	0.805 (0.576 to 1.125)	0.893 (0.759 to 1.051)	0.778 (0.591 to 1.025)
	50	0.547 (0.253 to 1.184)	0.792 (0.525 to 1.194)	0.586 (0.319 to 1.078)
T12 to T24	−50	0.145 (0.015 to 1.412)	1.011 (0.626 to 1.632)	1.722 (0.955 to 3.107)
	−25	0.466 (0.189 to 1.150)	1.010 (0.844 to 1.207)	1.517 (0.984 to 2.340)
	−9	0.806 (0.620 to 1.047)	1.006 (0.944 to 1.072)	1.128 (1.011 to 1.259)
	0	Ref.	Ref.	Ref.
	9	1.105 (0.930 to 1.312)	0.990 (0.939 to 1.045)	0.929 (0.852 to 1.013)
	25	1.065 (0.723 to 1.570)	0.961 (0.781 to 1.183)	0.769 (0.502 to 1.179)
	50	0.814 (0.293 to 2.260)	0.936 (0.657 to 1.332)	0.672 (0.329 to 1.371)

Estimates were created using restricted cubic spline regressions with three knots placed at fixed percentiles (10%, 50%, and 90%) of the physical activity variable, and 0 MET-hours was chosen as the reference category. 3, 9, 25, 50, and 70 MET-hrs correspond to the 15^th^, 25^th^, 50^th^, 75^th^ and 85^th^ percentile in the general population. The 15^th^, 25^th^, 50^th^ and 75^th^ percentile were also used to calculate HRs for change between subsequent timepoints.

Bold values illustrate statistically significant associations.

Note that the number of deaths is below the number of variables divided by 10 for T12 to T24 for both surgery only CRC and mCRC analyses, possibly resulting in unstable estimates.

Abbreviations: CRC = colorectal cancer; PA = physical activity; HR = hazard ratio; CI = confidence interval; MET-hrs/wk = metabolic equivalent of task-hours per week; T0 = diagnosis; T6 = 6 months after diagnosis; T12 = 12 months after diagnosis; T24 = 24 months after diagnosis.

In (neo-)adjuvantly treated CRC patients, both moderate and high vs low PA were associated with improved OS at T0, T12, and T24, with continuous associations also significant for those timepoints. At T12, the pattern was nonlinear, with limited survival differences above ∼50 MET-hrs/wk. Compared to remaining inactive, changing PA from inactive to active was associated with improved OS between T6 and T12 (HR = 0.53, 955 CI = 0.31 to 0.90), as was remaining active between both T6 and T12 (HR = 0.63, 95% CI = 0.43 to 0.93), and T12 and T24 (HR = 0.40, 95% CI =  0.25 to 0.64). Continuous PA changes showed no significant associations, and no significant nonlinear patterns were observed.

In mCRC patients, moderate vs low PA was associated with improved OS at T0 and T12, as was high vs low PA at T0, T12 and T24. Continuous associations were significant at all timepoints except T6. At T0, the pattern was nonlinear with limited survival differences above ∼40 MET-hrs/wk. Compared to remaining inactive, changing PA from active to inactive was associated with improved survival between T0 and T6 (HR = 0.61, 95% CI =  0.39 to 0.95), as was changing from inactive to active between T6 and T12 (HR = 0.53, 95% CI =  0.29 to 0.99). Continuous PA change was associated with improved OS between T6 and T12 (HR per 1 SD increase = 0.79, 95% CI =  0.66 to 1.00).

### Supplemental analyses

Analyses of total PA, MVPA and guideline adherence yielded generally comparable results (Figures S2–S7, Tables S2–S3). Pooled stage I-III and I-IV analyses revealed consistent statistically significant associations at all timepoints, with nonlinear patterns except at T24. Consistent associations were also observed for remaining active vs remaining inactive across all subsequent timepoints (Figures S8–S11, Tables S4–S5).

### Sensitivity analyses

**Figure 4.**
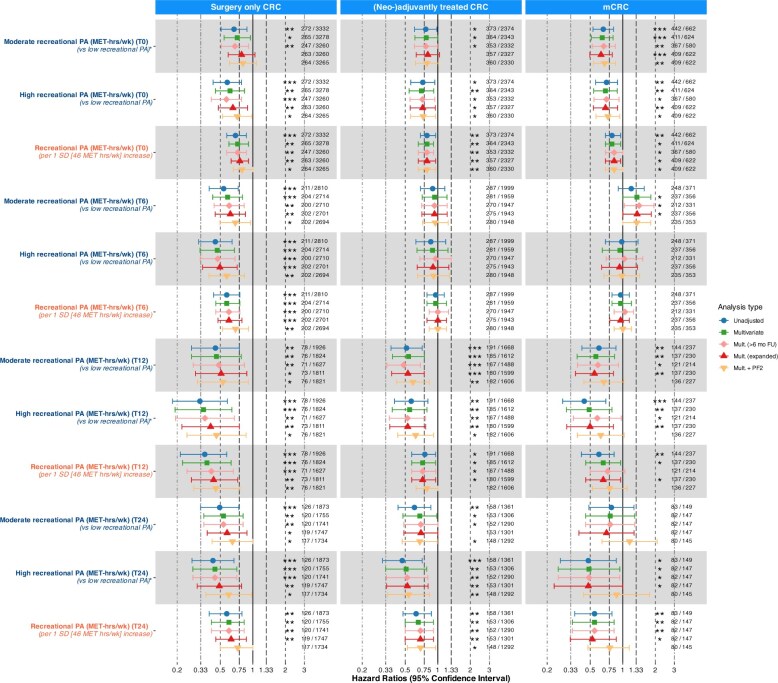
Timepoint sensitivity analyses of Cox proportional hazard models for recreational physical activity and overall survival, with varying covariate adjustments and follow-up restrictions. PA categorization was based on cut-offs from tertiles created in an age- and sex-matched sample of the general population. Low: tertile 1, moderate: tertile 2, high: tertile 3. Standard deviations were also calculated based on the general population sample. Numbers depict deaths/total patients in analysis. Stars indicate significant associations (*: *P* = .01 to <.05; **: *P* = .001 to <.01; *** *P* <.001). Fixed covariates: age (continuous), sex (male, female), primary tumor site (colon, rectum), cohort (PLCRC, COLON). Covariates at time of PA measurement: BMI (18.5-25, other), stoma (yes, no). Additional covariates in stage IV analyses: number of metastases (1, >1), liver-only metastasis (yes, no), surgery of primary tumor (yes, no), metastasectomy (yes, no), additional treatment during first disease episode (none, chemotherapy, radiotherapy, both). Unadjusted models included only PA as exposure. Mult. (≥6 mo FU): restriction to patients with ≥6 months FU after last used PA questionnaire. Mult. (expanded): further adjustment for smoking status (current, prior, former), education (low, moderate, high), marital status (single, married/in-law). Mult. + PF: further adjustment for functional status using the PF2 subscale of the EORTC QLQ-C30 at the time of PA assessment (dichotomized at >66.7 vs. ≤66.7). Abbreviations: CRC = colorectal cancer; PA = physical activity; HR = hazard ratio; CI = confidence interval; MET-hrs/wk = metabolic equivalent of task-hours per week; SD = standard deviation; PF = physical functioning; EORTC QLQ-C30 = European Organization for the Research and Treatment of Cancer Quality of Life Questionnaire-Core 30.

**Figure 5.**
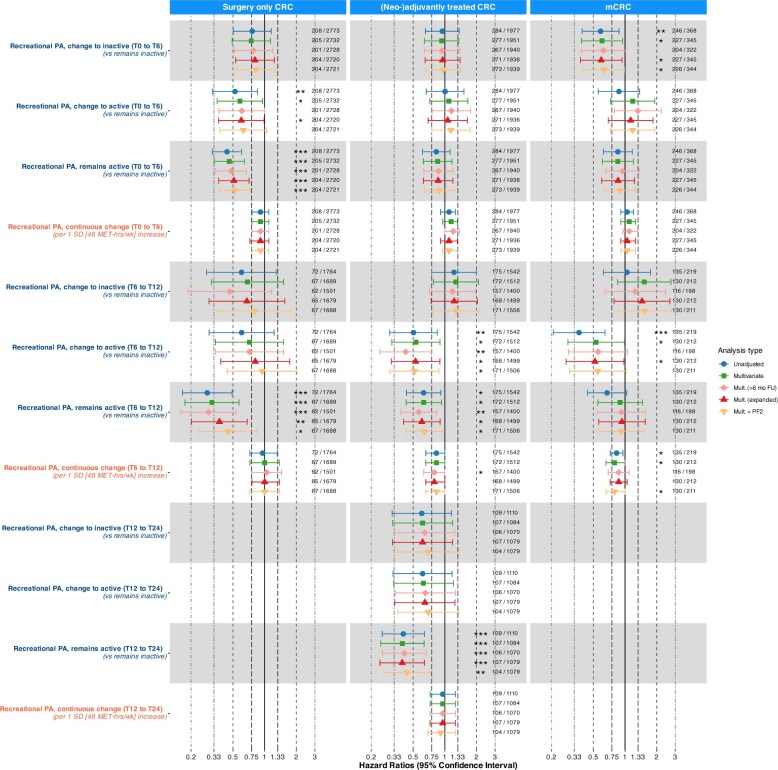
Changes between subsequent timepoint sensitivity analyses of Cox proportional hazard models for recreational physical activity and overall survival, with varying covariate adjustments and follow-up restrictions. PA categorization was based on cut-offs from tertiles created in an age- and sex-matched sample of the general population. Low: tertile 1, moderate: tertile 2, high: tertile 3. Remains inactive: tertile 1 at both timepoints; change to inactive: tertile 2/3 at first- and tertile 1 at second timepoint; change to active: tertile 1 at first- and tertile 2/3 at second timepoint; remains active: tertile 2/3 at both timepoints. Standard deviations were also calculated based on the general population sample. T12 to T24 change analyses were removed for surgery only CRC and stage IV CRC due to limited events. Numbers depict deaths/total patients in analysis. Stars indicate significant associations (*: *P* = .01 to <.05; **: *P* = .001 to <.01; *** *P* <.001). Fixed covariates: age (continuous), sex (male, female), primary tumor site (colon, rectum), cohort (PLCRC, COLON). Covariates at time of PA measurement: BMI (18.5-25, other), stoma (yes, no). Additional covariates in stage IV analyses: number of metastases (1, >1), liver-only metastasis (yes, no), surgery of primary tumor (yes, no), metastasectomy (yes, no), additional treatment during first disease episode (none, chemotherapy, radiotherapy, both). Unadjusted models included only PA as exposure. Mult. (≥6 mo FU): restriction to patients with ≥6 months FU after last used PA questionnaire. Mult. (expanded): further adjustment for smoking status (current, prior, former), education (low, moderate, high), marital status (single, married/in-law). Mult. + PF: further adjustment for functional status using the PF2 subscale of the EORTC QLQ-C30 at the time of first of the 2 subsequent PA assessments (dichotomized at >66.7 vs. ≤66.7). Abbreviations: CRC = colorectal cancer; PA = physical activity; HR = hazard ratio; CI = confidence interval; MET-hrs/wk = metabolic equivalent of task-hours per week; SD = standard deviation; PF = physical functioning; EORTC QLQ-C30 = European Organization for the Research and Treatment of Cancer Quality of Life Questionnaire-Core 30.

Effect estimates were broadly consistent with the main models, shifting slightly towards the null after adjustment for sociodemographic determinants and more markedly after adjustment for physical functioning (Figures 4–5, Tables S9–S10). Of 64 PA estimates tested in the main analysis, 39 were significant. Excluding patients with <6 months of follow-up rendered 5/39 associations non-significant and 8/64 estimates changed >10%, none of which changed significance. Adjustment for sociodemographic determinants rendered 4/39 associations non-significant and 10/64 estimates changed >10%, none of which changed significance. Adjustment for physical functioning rendered 12/39 associations non-significant and 27/64 estimates changed >10%, including 7 that changed significance.

Patients completing only 1 questionnaire reported less recreational PA at diagnosis compared to those who returned ≥3 questionnaires (Table S6). Assigning missing PA values as active did not alter the significance of associations (Figure S12, Tables S7–S8). Assigning missing PA values as inactive weakened some associations, mainly at later timepoints with high missingness, but overall direction of associations remained.

## Discussion

In this large CRC cohort, higher recreational PA up to 2 years post-diagnosis was associated with improved survival across all subgroups of stage I-IV patients, except at 6 months after diagnosis for (neo-)adjuvantly treated and mCRC patients. Several associations showed a non-linear inverse relationship, with survival gains plateauing near 50 MET-hrs/wk, translating to ∼10 hours of moderate-to-vigorous-intensity recreational PA. This aligns with the most active 25% in the age- and sex-matched general population. Becoming or remaining active was significantly associated with prolonged survival, especially from 6 months to 12 and 24 months after diagnosis. For surgery-only patients, this association was already significant from diagnosis to 6 months later. Estimates were largely robust across sensitivity analyses; however, some associations lost statistical significance, particularly after adjustment for physical functioning, suggesting that part of the observed benefit of PA may operate through maintained functional status.

Our single-timepoint findings generally align with previous studies.^3^ We observed no association between PA and OS at 6 months post-diagnosis among (neo-)adjuvantly treated CRC and mCRC patients. Prior work did not include subgroup analyses or PA data at this timepoint, limiting comparability. For PA change and OS, 2 studies reported significant associations when the second PA measure was taken ≥1 year post-diagnosis.^10^^,^  ^12^ One also examined PA change 6 months after diagnosis, reporting no significant association,^12^ similar to our findings for (neo-)adjuvantly treated and mCRC patients, with our results even showing a significant association with prolonged survival in mCRC patients who changed from active to inactive. This may reflect the short-term negative impact of cancer treatments on PA, while having a lasting impact on prognosis.^18^^,^  ^29^ Fewer patients maintained high PA at 6 months post-diagnosis in our data, supporting this interpretation. Healthier patients may also receive longer or more intensive treatment, temporarily reducing PA and potentially confounding survival associations; an effect that may be obscured when combining stage I–III or I–IV patients. Indeed, we observed significant associations at T6 in stage I–III/I–IV analyses, likely driven by surgery-only patients not undergoing treatment at that timepoint. In line with this, raw estimates tended to be stronger at later timepoints (T12-T24), supporting the idea that PA better reflects underlying health once initial treatment is completed. Reverse causation is also possible, with patients in poorer health less likely to remain active. Adjustment for physical functioning attenuated results, yet hazard ratios mostly remained (significantly) below 1, suggesting PA benefits beyond functional status alone. Several mechanisms have been proposed, including reduction of systemic inflammation, enhancement of anti-tumor immunity, and inhibition of metastatic potential,^30-33^ and a general survival benefit of improved cardiorespiratory fitness.^34^ Prospective studies with repeated PA assessments in the first year and more detailed longitudinal data on comorbidities, recurrence, and treatment are needed to further clarify these patterns.

To our knowledge, no previous original study has assessed the linearity of the relationship between PA and survival in CRC patients, focusing instead on categorical associations.^3^ Markozannes et al. reported an inverse association up to 20 MET-hrs/wk of recreational PA in a non-linear dose–response meta-analysis, contrasting with our plateau at ∼50 MET-hrs/wk. This discrepancy may reflect differences in PA questionnaires, as SQUASH typically yields higher values than other validated tools,^35-38^ as well as variation in PA patterns across countries.^39^ Indeed, studies using other questionnaires report a 75th percentile near 20 MET-hrs/wk, comparable to the 50 MET-hrs/wk 75th percentile in our data.^10^^,^  ^40^^,^^41^

Given questionnaire variability and our observational design, caution is warranted in applying an absolute cut-off. In particular, confounding factors such as comorbidities and ongoing treatments may influence the relationship between PA and survival. Randomized controlled trials are therefore crucial for both establishing causality, and determining the optimal dose and duration of PA interventions in the different CRC subgroups. A recent trial in patients with resected colon cancer demonstrated that a structured 3-year exercise program initiated after adjuvant chemotherapy significantly improved disease-free survival,^13^ underscoring the potential of sustained interventions in defined subgroups.

### Strengths & limitations

Strengths include combining two large prospective CRC cohorts, enabling repeated PA assessment and subgroup analyses in substantial populations. Extensive supplemental analyses were performed to enhance comparability with other studies, and sensitivity analyses were conducted to address the impact of missing data and robustness of estimates when including additional variables.

Limitations include the observational design and potential residual confounding. Notably missing were data on toxicity, treatment compliance, recurrence, and comorbidities during follow-up; factors potentially affecting both PA and survival. Information on cancer-specific survival was also not available.

## Conclusion

Moderate-to-high-intensity recreational PA is associated with improved survival in all CRC patient subgroups up to 2 years post-diagnosis, with some evidence suggesting smaller gains beyond ∼50 MET-hrs/week, corresponding to the highest 25% of the population. Notably, transitioning from inactivity to activity was significantly associated with improved survival during the early months after diagnosis for surgery-only patients, and later for those undergoing (neo-)adjuvant therapy or with metastatic disease. These observational findings can inform the design of randomized clinical trials and help strengthen emerging trial evidence on the causal relationship between changes in PA and overall survival.

## Supplementary Material

pkaf116_Supplementary_Data

## Data Availability

The data underlying this article were provided by the Netherlands Cancer Registry (NCR) by permission. Data will be shared on request to the corresponding author with permission of the NCR (https://iknl.nl/en/ncr/apply-for-data).
