# Supplementary material for: Physical activity patterns after diagnosis and survival of prognostic colorectal cancer subgroups
Source: JNCI Cancer Spectr. 2025 Dec 15;10(1):pkaf116. doi: 10.1093/jncics/pkaf116 (PMC12783896; doi:10.1093/jncics/pkaf116)
Supplement: pkaf116_Supplementary_Data [file pkaf116_supplementary_data.zip › Supplemental data - CRC PA change & survival v2.pdf]

Supplementary material

Table S1 – Study characteristics.......... 3

Table S2 – Associations from Cox proportional hazard models for timepoint associations of total physical activity (MET-hours per week), moderate and vigorous physical activity (MET-hours per week) and adherence to PA guidelines with overall survival 4

Table S3 – Associations from Cox proportional hazard models for change between subsequent timepoint associations of total physical activity (MET-hours per week), total moderate and vigorous physical activity (MVPA, MET-hours per week), and adherence to physical activity guidelines with overall survival 6

Table S4 – Associations from Cox proportional hazard models for timepoint associations of recreational physical activity (MET-hours per week) with overall survival for stage I to III and stage I to IV colorectal cancer patients. 8

Table S5 – Associations from Cox proportional hazard models for change between subsequent timepoints associations of recreational physical activity (MET-hours per week) with overall survival for stage I to III and stage I to IV CRC patients. 9

Table S6 – Physical activity characteristics of colorectal cancer patients at diagnosis who either returned 1 or at least 3 SQUASH questionnaires. 10

Table S7 – Timepoint sensitivity analyses of Cox proportional hazard models for overall survival, with missing recreational physical activity (MET-hours/week) revalued as low or high.” 11

Table S8 – Changes between subsequent timepoint sensitivity analyses of Cox proportional hazard models for overall survival, with missing recreational physical activity (MET-hours/week) revalued as low or high. 12

Table S9 – Timepoint sensitivity analyses of Cox proportional hazard models for recreational physical activity and overall survival, with varying covariate adjustments and follow-up restrictions. 14

Table S10 – Changes between subsequent timepoint sensitivity analyses of Cox proportional hazard models for recreational physical activity and overall survival, with varying covariate adjustments and follow-up restrictions. 17

Fig S1 – Recreational physical activity descriptives over time 20

Fig S2 – Associations from Cox proportional hazard models for timepoint associations of total physical activity (MET-hours per week), moderate and vigorous physical activity (MVPA, MET-hours per week) and adherence to PA guidelines with overall survival 21

Fig S3 – Restricted cubic splines from Cox proportional hazard models for timepoint associations of total physical activity (MET-hours per week) with overall survival 22

Fig S4 – Restricted cubic splines from Cox proportional hazard models for timepoint associations of total moderate and vigorous physical activity (MVPA, MET-hours per week) with overall survival 23

Fig S5 – Associations from Cox proportional hazard models for change between subsequent timepoint associations of total physical activity (MET-hours per week), total moderate and vigorous physical activity (MVPA, MET-hours per week), and adherence to physical activity guidelines with overall survival 24

Data supplement, Fig 6 – Restricted cubic splines from Cox proportional hazard models for change between subsequent timepoints associations of total physical activity (MET-hours per week) with overall survival 25

Fig S7 – Restricted cubic splines from Cox proportional hazard models for change between subsequent timepoints associations of total moderate and vigorous physical activity (MVPA, MET-hours per week) 26

Fig S8 – Associations from Cox proportional hazard models for timepoint associations of recreational physical activity (MET-hours per week) with overall survival for stage I to III and stage I to IV colorectal cancer patients. 27

Fig S9 – Restricted cubic splines from Cox proportional hazard models for timepoint associations of recreational physical activity (MET-hours per week) with overall survival for stage I to III and stage I to IV CRC patients....................................... 28

Fig S10 – Associations from Cox proportional hazard models for change between subsequent timepoints associations of recreational physical activity (MET-hours per week) with overall survival for stage I to III and stage I to IV CRC patients. 29

Fig S11 – Restricted cubic splines from Cox proportional hazard models for change between subsequent timepoints associations of recreational physical activity (MET-hours per week) with overall survival for stage I to III and stage I to IV CRC patients 30

Fig S12 – Sensitivity analyses where missing PA values are revalued to either low or high for recreational physical activity (MET-hours/week) 31

Table S1 – Study characteristics

	Cohort 1: PLCRC	Cohort 2: COLON
**Cohort enrolment**	2013 – present (63 hospitals)	2010 – 2020 (11 hospitals)
**Inclusion criteria**	Histological proof of colorectal, small bowel or anal cancer, or a strong suspicion after imaging. Patients can be recruited at any point in time after first diagnosis.	Newly diagnosed colorectal cancer (ICD codes C18-20).
**Exclusion criteria**	<18 years, non-Dutch speaking patients, mentally incompetent patients.	<18 years, non-Dutch speaking patients, patients with a history of CRC, (partial) bowel resection, chronic inflammatory bowel disease, hereditary CRC syndromes (e.g. Lynch syndrome, Familial Adenomatous Polyposis, Peutz-Jegher), dementia or another mental condition obstructing participation
**Cancer and treatment information**	Netherlands cancer registry. Available for the primary diagnosis (i.e., first disease episode).<br>Information on (treatment) of) possible recurrence and/or progression of disease was not available for the majority of patients and therefore not used in the current analyses.	Netherlands cancer registry. Available for the primary diagnosis (i.e., first disease episode).<br>Information on (treatment) of) possible recurrence and/or progression of disease was not available for the majority of patients and therefore not used in the current analyses.
**Physical activity assessment**	SQUASH questionnaire[1]	SQUASH questionnaire[1]
**Questionnaire timepoints**	At diagnosis (T0) and, 6, 12 and 24 months after diagnosis (T6, T12, T24)	At diagnosis (T0) and, 6, 12 and 24 months after diagnosis (T6, T12, T24).<br>For T12, only patients receiving adjuvant treatment received questionnaires.
**Patients selected**	2016 February – 2022 December (n = 14820)	2010 August – 2020 February (n = 2104)
**Additional exclusion criterium**	Additional informed consent for receiving questionnaires. (n = 2792 excluded)<br>T0 SQUASH >60 days after diagnosis. (n = 6246 excluded)	All patients provide informed consent for receiving questionnaires at inclusion.<br>T0 SQUASH >60 days after diagnosis or missing date of first questionnaire.<br>(n = 373 excluded)
**Included in this study**	All patients: n = 5782<br>Surgery only CRC: n = 2603;<br>(Neo-)adjuvant CRC: n = 1839<br>mCRC: n = 542	All patients: n = 1731<br>Surgery only CRC: n = 792<br>(Neo-)adjuvant CRC: n = 567<br>mCRC: n = 127
**Censoring date**	February 1st 2024	February 1st 2024

Abbreviations: PLCRC, prospective Dutch colorectal cancer cohort; COLON, Colorectal cancer, Observational, LONngitudinal study; ICD, international classification of diseases, CRC, colorectal cancer; PA, physical activity; MET-hrs/wk, metabolic equivalent of task-hours per week.

Table S2 – Associations from Cox proportional hazard models for timepoint associations of total physical activity (MET-hours per week), moderate and vigorous physical activity (MET-hours per week) and adherence to PA guidelines with overall survival

		Surgery only CRC			(Neo-)adjuvantly treated CRC			mCRC		
**Time**	**Category**	**FU (IQR)**	**Death/total**	**HR (95% CI)**	**FU (IQR)**	**Death/total**	**HR (95% CI)**	**FU (IQR)**	**Death/total**	**HR (95% CI)**
**Total PA (MET-hrs/wk)**										
T0	Low	47 (28-73)	170/1367	Ref.	50 (29-75)	167/865	Ref.	20 (11-35)	195/266	Ref.
T0	Moderate	43 (27-69)	66/1142	**0.678 (0.506 - 0.908)**	46 (27-71)	126/850	0.974 (0.768 - 1.236)	27 (16-42)	133/211	0.830 (0.660 - 1.044)
T0	High	39 (26-63)	29/769	**0.527 (0.352 - 0.788)**	43 (27-67)	71/629	0.818 (0.613 - 1.092)	29 (19-45)	83/147	**0.623 (0.477 - 0.813)**
T0	Continuous	43 (27-69)	265/3278	**Non-linear, significant**	46 (28-71)	364/2344	**0.849 (0.720 - 1.000)**	24 (14-40)	411/624	**0.782 (0.663 - 0.921)**
T6	Low	52 (31-84)	138/1183	Ref.	56 (33-85)	179/1057	Ref.	33 (20-47)	167/238	Ref.
T6	Moderate	47 (28-74)	44/963	**0.574 (0.404 - 0.815)**	48 (29-68)	77/597	0.988 (0.752 - 1.299)	35 (22-52)	51/82	0.850 (0.614 - 1.176)
T6	High	39 (25-62)	22/568	0.649 (0.407 - 1.035)	38 (27-60)	25/306	0.762 (0.496 - 1.170)	34 (24-50)	19/36	**0.582 (0.354 - 0.956)**
T6	Continuous	47 (28-74)	204/2714	**Non-linear, significant**	50 (30-76)	281/1960	0.921 (0.720 - 1.085)	33 (21-49)	237/356	**0.782 (0.610 - 1.000)**
T12	Low	39 (25-54)	58/688	Ref.	52 (32-77)	97/651	Ref.	35 (24-48)	98/138	Ref.
T12	Moderate	36 (24-52)	15/693	**0.356 (0.200 - 0.633)**	46 (28-68)	59/611	0.828 (0.594 - 1.154)	39 (27-58)	31/66	0.784 (0.508 - 1.211)
T12	High	36 (24-55)	3/443	**0.128 (0.040 - 0.412)**	43 (28-62)	29/351	0.787 (0.514 - 1.207)	43 (34-63)	8/26	**0.416 (0.199 - 0.866)**
T12	Continuous	37 (24-54)	76/1824	**0.266 (0.161 - 0.439)**	48 (29-69)	185/1613	**Non-linear, significant**	36 (26-52)	137/230	**0.562 (0.404 - 0.782)**
T24	Low	63 (40-99)		Ref.	65 (41-92)	92/535	Ref.	43 (34-63)	60/86	Ref.
T24	Moderate	57 (38-84)	28/622	**0.637 (0.410 - 0.990)**	57 (40-76)	43/493	**0.680 (0.470 - 0.984)**	51 (42-61)	17/38	**0.504 (0.281 - 0.905)**
T24	High	56 (38-79)	11/384	**0.488 (0.256 - 0.931)**	56 (38-79)	18/279	**0.539 (0.323 - 0.901)**	64 (48-78)	5/23	**0.228 (0.088 - 0.591)**
T24	Continuous	59 (39-88)	81/749	**0.476 (0.342 - 0.720)**	60 (40-84)	153/1307	**0.610 (0.439 - 0.782)**	48 (36-67)	82/147	**Non-linear, significant**
**Total MVPA (MET-hrs/wk)**										
T0	Low	48 (29-72)	126/1067	Ref.	49 (29-75)	148/722	Ref.	20 (11-34)	160/219	Ref.
T0	Moderate	42 (26-67)	81/1216	**0.669 (0.502 - 0.891)**	44 (27-69)	128/871	0.797 (0.627 - 1.012)	25 (14-40)	141/218	0.900 (0.713 - 1.137)
T0	High	40 (26-67)	58/995	**0.608 (0.442 - 0.837)**	45 (27-69)	88/750	**0.648 (0.496 - 0.847)**	27 (18-43)	110/187	**0.636 (0.494 - 0.818)**
T0	Continuous	43 (27-69)	265/3278	**Non-linear, significant**	46 (28-71)	364/2343	**Non-linear, significant**	24 (14-40)	411/624	**Non-linear, significant**
T6	Low	53 (32-84)	111/889	Ref.	56 (33-84)	140/814	Ref.	31 (18-46)	131/183	Ref.
T6	Moderate	46 (28-72)	56/1029	**0.543 (0.391 - 0.753)**	49 (30-74)	100/704	0.993 (0.765 - 1.291)	36 (24-54)	71/116	0.900 (0.663 - 1.220)
T6	High	40 (26-65)	37/796	**0.488 (0.333 - 0.717)**	40 (27-62)	41/441	0.777 (0.542 - 1.114)	32 (22-44)	35/57	**0.649 (0.436 - 0.966)**
T6	Continuous	47 (28-74)	204/2714	**Non-linear, significant**	50 (30-76)	281/1959	0.932 (0.755 - 1.150)	33 (21-49)	237/356	0.810 (0.656 - 1.000)
T12	Low	38 (25-54)	44/497	Ref.	52 (34-75)	81/488	Ref.	34 (23-46)	72/102	Ref.
T12	Moderate	37 (24-54)	23/714	**0.453 (0.268 - 0.764)**	46 (27-68)	65/650	0.720 (0.517 - 1.002)	38 (30-53)	48/88	**0.596 (0.404 - 0.879)**
T12	High	35 (24-53)	9/613	**0.180 (0.086 - 0.376)**	45 (30-66)	39/474	**0.592 (0.401 - 0.873)**	39 (31-58)	17/40	**0.564 (0.327 - 0.973)**
T12	Continuous	37 (24-54)	76/1824	**0.280 (0.170 - 0.461)**	48 (29-69)	185/1612	**Non-linear, significant**	36 (26-52)	137/230	**0.570 (0.400 - 0.869)**
T24	Low	62 (40-94)	60/536	Ref.	61 (40-86)	73/414	Ref.	46 (34-67)	42/63	Ref.
T24	Moderate	59 (39-92)	41/676	**0.612 (0.406 - 0.922)**	60 (40-85)	53/519	**0.637 (0.444 - 0.914)**	42 (36-60)	28/49	0.661 (0.387 - 1.128)
T24	High	56 (37-80)	19/543	**0.402 (0.235 - 0.686)**	57 (39-79)	27/373	**0.483 (0.308 - 0.757)**	60 (45-74)	12/35	**0.308 (0.155 - 0.611)**
T24	Continuous	59 (39-88)	120/1755	**Non-linear, significant**	60 (40-84)	153/1306	**Non-linear, significant**	48 (36-67)	82/147	**0.430 (0.261 - 0.656)**

PA guideline											
	T0	Non-adherence	46 (28-72)	197/1921	Ref.	48 (28-74)	238/1335	Ref.	23 (13-38)	271/388	Ref.
	T0	Adherence	40 (26-65)	68/1357	**0.620 (0.468 - 0.821)**	44 (27-68)	126/1008	**0.797 (0.641 - 0.991)**	27 (17-42)	140/236	**0.754 (0.611 - 0.931)**
	T6	Non-adherence	50 (31-80)	162/1603	Ref.	54 (33-82)	199/1267	Ref.	32 (20-48)	175/259	Ref.
	T6	Adherence	41 (26-67)	41/1093	**0.467 (0.329 - 0.663)**	44 (27-64)	80/682	1.051 (0.804 - 1.374)	34 (24-51)	62/97	0.999 (0.741 - 1.349)
	T12	Non-adherence	38 (25-54)	58/960	Ref.	50 (32-73)	131/918	Ref.	35 (24-49)	99/156	Ref.
	T12	Adherence	36 (24-53)	17/849	**0.417 (0.240 - 0.725)**	44 (27-65)	53/685	**0.652 (0.472 - 0.900)**	40 (31-57)	38/73	0.843 (0.573 - 1.240)
	T24	Non-adherence	62 (41-97)	99/1028	Ref.	61 (41-86)	105/747	Ref.	48 (36-67)	56/95	Ref.
	T24	Adherence	55 (37-77)	21/715	**0.404 (0.251 - 0.652)**	57 (38-81)	47/556	0.723 (0.511 - 1.024)	49 (36-64)	26/52	0.651 (0.390 - 1.086)

Effect estimates for continuous activity are shown per 1 SD increase (82 MET-hrs/wk for total PA, 70 MET-hrs/wk for total MVPA).
PA categorization was based on cut-offs from tertiles created in an age- and sex-matched sample of the general population. Low: tertile 1, moderate: tertile 2, high: tertile 3.
Abbreviations: CRC, colorectal cancer; FU, FU time in months; IQR, interquartile range; HR, hazard ratio; CI, confidence interval; MVPA, moderate and vigorous physical activity; MET-hrs/wk, metabolic equivalent of task-hours per week.

Table S3 – Associations from Cox proportional hazard models for change between subsequent timepoint associations of total physical activity (MET-hours per week), total moderate and vigorous physical activity (MVPA, MET-hours per week), and adherence to physical activity guidelines with overall survival

		Surgery only CRC			(Neo-)adjuvantly treated CRC			mCRC		
Time	Category	FU (IQR)	Death/total	HR (95% CI)	FU (IQR)	Death/total	HR (95% CI)	FU (IQR)	Death/total	HR (95% CI)
**Total PA (MET-hrs/wk)**										
T0 to T6	Remains inactive	51 (30-85)	107/762	Ref.	57 (33-85)	100/538	Ref.	30 (17-42)	70/100	Ref.
T0 to T6	Change to inactive	48 (29-77)	34/441	0.735 (0.495 - 1.091)	55 (32-83)	78/517	1.016 (0.749 - 1.378)	34 (22-52)	88/129	1.041 (0.742 - 1.460)
T0 to T6	Change to active	43 (28-70)	23/341	0.639 (0.403 - 1.014)	43 (29-65)	23/170	0.987 (0.624 - 1.561)	39 (19-47)	16/22	1.339 (0.745 - 2.408)
T0 to T6	Remains active	41 (26-66)	41/1188	**0.452 (0.308 - 0.663)**	43 (27-63)	76/726	0.875 (0.640 - 1.196)	34 (23-52)	53/94	0.707 (0.485 - 1.033)
T0 to T6	Continuous change	45 (28-72)	205/2732	0.921 (0.782 - 1.085)	50 (30-75)	277/1951	1.000 (0.849 - 1.178)	33 (21-49)	227/345	1.000 (0.849 - 1.178)
T6 to T12	Remains inactive	37 (24-52)	44/430	Ref.	55 (33-80)	72/468	Ref.	34 (24-43)	67/95	Ref.
T6 to T12	Change to inactive	39 (26-57)	9/208	**0.411 (0.198 - 0.856)**	51 (30-71)	15/149	0.703 (0.402 - 1.229)	38 (24-49)	26/35	1.207 (0.725 - 2.008)
T6 to T12	Change to active	38 (27-55)	5/228	**0.254 (0.098 - 0.655)**	53 (32-77)	31/317	0.710 (0.463 - 1.088)	44 (34-63)	19/38	0.794 (0.455 - 1.386)
T6 to T12	Remains active	35 (24-52)	9/823	**0.154 (0.074 - 0.322)**	41 (27-62)	54/578	0.876 (0.606 - 1.266)	36 (25-58)	18/44	0.577 (0.326 - 1.020)
T6 to T12	Continuous change	37 (24-53)	67/1689	Non-linear, non-significant	49 (30-71)	172/1512	0.849 (0.720 - 1.085)	36 (27-52)	130/212	0.849 (0.663 - 1.085)
T12 to T24	Remains inactive	45 (35-59)	18/273	Ref.	65 (43-87)	46/283	Ref.	42 (34-55)	29/41	Ref.
T12 to T24	Change to inactive	43 (34-62)	3/150	NA	50 (34-73)	18/136	1.115 (0.640 - 1.944)	50 (42-59)	8/16	NA
T12 to T24	Change to active	50 (39-60)	6/131	NA	56 (40-79)	11/162	**0.489 (0.251 - 0.952)**	52 (37-73)	10/19	NA
T12 to T24	Remains active	42 (33-58)	8/550	NA	55 (37-72)	32/503	**0.540 (0.336 - 0.869)**	58 (44-65)	7/30	NA
T12 to T24	Continuous change	44 (34-59)	35/1104	NA	57 (38-78)	107/1084	**0.720 (0.518 - 0.921)**	48 (36-63)	54/106	NA
**Total MVPA (MET-hrs/wk)**										
T0 to T6	Remains inactive	51 (31-84)	82/568	Ref.	54 (33-85)	76/398	Ref.	30 (18-45)	53/78	Ref.
T0 to T6	Change to inactive	53 (31-78)	33/340	0.749 (0.496 - 1.131)	57 (34-83)	65/420	0.852 (0.610 - 1.190)	30 (19-47)	72/99	0.963 (0.659 - 1.407)
T0 to T6	Change to active	45 (29-73)	17/272	**0.543 (0.321 - 0.921)**	48 (30-71)	26/183	0.941 (0.599 - 1.477)	33 (23-56)	20/28	0.900 (0.530 - 1.529)
T0 to T6	Remains active	41 (26-67)	73/1552	**0.421 (0.302 - 0.587)**	44 (28-67)	110/950	0.789 (0.583 - 1.070)	35 (24-51)	82/140	0.747 (0.514 - 1.084)
T0 to T6	Continuous change	45 (28-72)	205/2732	0.869 (0.704 - 1.072)	50 (30-75)	277/1951	1.072 (0.932 - 1.233)	33 (21-49)	227/345	1.072 (0.869 - 1.233)
T6 to T12	Remains inactive	39 (25-54)	28/300	Ref.	56 (35-81)	51/330	Ref.	33 (24-43)	48/69	Ref.
T6 to T12	Change to inactive	37 (24-53)	9/162	0.769 (0.355 - 1.666)	43 (26-64)	21/132	1.278 (0.765 - 2.135)	39 (27-55)	21/27	1.211 (0.701 - 2.090)
T6 to T12	Change to active	40 (26-59)	10/184	0.628 (0.297 - 1.326)	53 (32-76)	31/263	0.850 (0.542 - 1.332)	44 (31-61)	20/35	**0.568 (0.326 - 0.990)**
T6 to T12	Remains active	35 (24-52)	20/1043	**0.249 (0.136 - 0.458)**	45 (28-67)	69/787	0.735 (0.506 - 1.068)	36 (27-53)	41/81	0.727 (0.463 - 1.142)
T6 to T12	Continuous change	37 (24-53)	67/1689	Non-linear, non-significant	49 (30-71)	172/1512	0.810 (0.656 - 1.000)	36 (27-52)	130/212	0.869 (0.656 - 1.150)
T12 to T24	Remains inactive	48 (36-60)	15/195	Ref.	62 (43-86)	30/196	Ref.	46 (35-77)	16/26	Ref.
T12 to T24	Change to inactive	42 (33-59)	4/115	NA	51 (34-64)	21/122	1.558 (0.886 - 2.740)	47 (36-56)	11/18	NA
T12 to T24	Change to active	48 (34-60)	4/95	NA	58 (40-83)	14/138	0.680 (0.359 - 1.286)	48 (39-86)	8/13	NA
T12 to T24	Remains active	43 (34-59)	12/699	NA	57 (38-76)	42/628	**0.493 (0.306 - 0.795)**	51 (37-62)	19/49	NA
T12 to T24	Continuous change	44 (34-59)	35/1104	NA	57 (38-78)	107/1084	0.869 (0.656 - 1.150)	48 (36-63)	54/106	NA
**PA guideline**										
T0 to T6	Remains inactive	50 (30-79)	137/1276	Ref.	53 (33-82)	152/900	Ref.	32 (18-46)	117/172	Ref.

	T0 to T6	Change to inactive	44 (28-75)	27/356	0.817 (0.538 - 1.240)	53 (31-80)	47/367	0.789 (0.568 - 1.096)	33 (23-48)	50/78	0.758 (0.532 - 1.079)
	T0 to T6	Change to active	39 (26-64)	12/299	**0.447 (0.247 - 0.811)**	45 (26-61)	22/195	0.968 (0.614 - 1.525)	35 (24-47)	27/38	1.016 (0.656 - 1.573)
	T0 to T6	Remains active	40 (26-67)	29/801	**0.437 (0.290 - 0.658)**	42 (28-64)	56/489	0.926 (0.677 - 1.266)	34 (27-54)	33/57	0.826 (0.554 - 1.233)
	T6 to T12	Remains inactive	38 (25-53)	45/703	Ref.	51 (33-78)	105/707	Ref.	35 (26-49)	78/125	Ref.
	T6 to T12	Change to inactive	35 (24-53)	6/192	0.597 (0.246 - 1.448)	47 (30-62)	14/147	0.820 (0.466 - 1.443)	35 (18-48)	16/20	**2.000 (1.097 - 3.644)**
	T6 to T12	Change to active	38 (25-53)	5/201	0.516 (0.202 - 1.317)	52 (30-75)	16/247	**0.481 (0.284 - 0.815)**	44 (35-60)	9/21	0.643 (0.314 - 1.317)
	T6 to T12	Remains active	35 (24-53)	10/577	**0.304 (0.150 - 0.613)**	43 (27-64)	36/401	0.787 (0.535 - 1.157)	35 (29-56)	27/46	1.338 (0.829 - 2.159)
	T12 to T24	Remains inactive	47 (35-59)	22/435	Ref.	60 (40-82)	64/497	Ref.	48 (36-68)	29/54	Ref.
	T12 to T24	Change to inactive	43 (33-58)	6/149	NA	52 (38-64)	10/116	0.824 (0.420 - 1.618)	50 (46-63)	5/12	NA
	T12 to T24	Change to active	44 (34-61)	0/123	NA	55 (38-78)	15/133	0.987 (0.559 - 1.741)	42 (37-62)	6/10	NA
	T12 to T24	Remains active	41 (33-58)	6/385	NA	56 (36-76)	17/336	**0.460 (0.268 - 0.788)**	49 (36-62)	14/30	NA

Effect estimates for continuous activity are shown per 1 SD increase (82 MET-hrs/wk for total PA, 70 MET-hrs/wk for total MVPA).
PA categorization was based on cut-offs from tertiles created in an age- and sex-matched sample of the general population. Low: tertile 1, moderate: tertile 2, high: tertile 3. Remains inactive: low at both timepoints; change to inactive: moderate/high at first- and low at second timepoint; change to active: low at first- and moderate/high at second timepoint; remains active: tertile 2/3 at both timepoints.
Abbreviations: CRC, colorectal cancer; FU, FU time in months; IQR, interquartile range; HR, hazard ratio; CI, confidence interval; MVPA, moderate and vigorous physical activity; MET-hrs/wk, metabolic equivalent of task-hours per week.

Table S4 – Associations from Cox proportional hazard models for timepoint associations of recreational physical activity (MET-hours per week) with overall survival for stage I to III and stage I to IV colorectal cancer patients.

			Stage I to III CRC			Stage I to IV CRC		
	Time	Category	FU (IQR)	Death/total	HR (95% CI)	FU (IQR)	Death/total	HR (95% CI)
	T0	Low	46 (28-73)	326/2037	Ref.	41 (25-70)	490/2263	Ref.
	T0	Moderate	46 (27-72)	271/2419	**0.771 (0.655 - 0.907)**	43 (26-69)	409/2642	**0.699 (0.612 - 0.798)**
	T0	High	41 (25-66)	208/2149	**0.689 (0.578 - 0.821)**	40 (24-65)	318/2326	**0.647 (0.561 - 0.746)**
	T0	Continuous	44 (27-70)	805/6605	**Non-linear, significant**	41 (25-68)	1217/7231	**Non-linear, significant**
	T6	Low	53 (31-83)	255/1689	Ref.	51 (30-81)	350/1835	Ref.
	T6	Moderate	50 (29-76)	221/2139	**0.813 (0.677 - 0.975)**	48 (28-74)	321/2282	**0.958 (0.821 - 1.116)**
	T6	High	42 (27-66)	126/1638	**0.684 (0.550 - 0.851)**	41 (27-65)	168/1706	**0.775 (0.642 - 0.935)**
	T6	Continuous	48 (28-75)	602/5466	**Non-linear, significant**	47 (28-74)	839/5823	**Non-linear, significant**
	T12	Low	41 (27-61)	131/1028	Ref.	40 (26-60)	189/1107	Ref.
	T12	Moderate	40 (26-62)	101/1582	**0.507 (0.390 - 0.658)**	40 (26-62)	154/1674	**0.544 (0.439 - 0.674)**
	T12	High	39 (26-58)	75/1366	**0.476 (0.357 - 0.636)**	39 (26-58)	101/1425	**0.468 (0.366 - 0.597)**
	T12	Continuous	40 (26-60)	307/3976	**Non-linear, significant**	40 (26-60)	444/4206	**Non-linear, significant**
	T24	Low	63 (40-92)	134/974	Ref.	63 (40-90)	168/1024	Ref.
	T24	Moderate	61 (40-87)	113/1386	**0.625 (0.486 - 0.804)**	60 (40-87)	146/1443	**0.640 (0.512 - 0.800)**
	T24	High	57 (38-81)	77/1180	**0.566 (0.426 - 0.752)**	56 (39-80)	92/1220	**0.507 (0.392 - 0.656)**
	T24	Continuous	60 (40-86)	324/3540	**Non-linear, significant**	60 (39-86)	406/3687	**0.630 (0.523 - 0.724)**

Effect estimates for continuous activity are shown per 1 SD increase (46 MET-hrs/wk).
PA categorization was based on cut-offs from tertiles created in an age- and sex-matched sample of the general population. Low: tertile 1, moderate: tertile 2, high: tertile 3.
Abbreviations: CRC, colorectal cancer; FU, FU time in months; IQR, interquartile range; HR, hazard ratio; CI, confidence interval; PA, physical activity; MET-hrs/wk, metabolic equivalent of task-hours per week.

Table S5 – Associations from Cox proportional hazard models for change between subsequent timepoints associations of recreational physical activity (MET-hours per week) with overall survival for stage I to III and stage I to IV CRC patients.

			Stage I to III CRC			Stage I to IV CRC		
	**Time**	**Category**	**FU (IQR)**	**Death/total**	**HR (95% CI)**	**FU (IQR)**	**Death/total**	**HR (95% CI)**
	T0 to T6	Remains inactive	50 (29-81)	154/989	Ref.	48 (28-79)	207/1066	Ref.
	T0 to T6	Change to inactive	53 (31-81)	100/719	0.860 (0.668 - 1.108)	53 (30-80)	139/785	**0.708 (0.569 - 0.881)**
	T0 to T6	Change to active	48 (31-74)	67/614	0.824 (0.618 - 1.100)	48 (30-73)	93/649	0.914 (0.714 - 1.169)
	T0 to T6	Remains active	44 (27-69)	277/3144	**0.694 (0.568 - 0.848)**	43 (27-68)	386/3312	**0.720 (0.607 - 0.854)**
	T0 to T6	Continuous change	47 (28-74)	598/5466	1.000 (0.912 - 1.148)	45 (28-72)	825/5812	1.047 (0.955 - 1.148)
	T6 to T12	Remains inactive	40 (27-62)	75/580	Ref.	39 (27-62)	106/623	Ref.
	T6 to T12	Change to inactive	40 (26-60)	41/367	0.955 (0.651 - 1.400)	40 (25-59)	65/399	1.109 (0.811 - 1.518)
	T6 to T12	Change to active	50 (30-70)	36/462	**0.511 (0.343 - 0.763)**	49 (30-69)	55/505	**0.485 (0.348 - 0.676)**
	T6 to T12	Remains active	39 (26-59)	128/2281	**0.496 (0.372 - 0.663)**	39 (26-59)	184/2375	**0.589 (0.461 - 0.753)**
	T6 to T12	Continuous change	40 (26-61)	280/3690	0.871 (0.758 - 1.047)	40 (26-60)	410/3902	**Non-linear, significant**
	T12 to T24	Remains inactive	51 (36-65)	42/356	Ref.	51 (36-65)	56/376	Ref.
	T12 to T24	Change to inactive	50 (35-65)	19/257	0.623 (0.360 - 1.079)	50 (35-65)	26/270	0.697 (0.435 - 1.120)
	T12 to T24	Change to active	51 (37-68)	18/251	0.584 (0.335 - 1.019)	51 (37-68)	23/258	0.684 (0.416 - 1.122)
	T12 to T24	Remains active	51 (35-66)	78/1626	**0.396 (0.270 - 0.579)**	51 (35-66)	106/1692	**0.437 (0.314 - 0.609)**
	T12 to T24	Continuous change	51 (36-66)	157/2490	1.000 (0.794 - 1.258)	51 (36-66)	211/2596	0.871 (0.724 - 1.047)

Effect estimates for continuous activity are shown per 1 SD increase (46 MET-hrs/wk)
PA categorization was based on cut-offs from tertiles created in an age- and sex-matched sample of the general population. Low: tertile 1, moderate: tertile 2, high: tertile 3. Remains inactive: low at both timepoints; change to inactive: moderate/high at first- and low at second timepoint; change to active: low at first- and moderate/high at second timepoint; remains active: tertile 2/3 at both timepoints.
Abbreviations: CRC, colorectal cancer; FU, FU time in months; IQR, interquartile range; HR, hazard ratio; CI, confidence interval; Ref., reference; PA, physical activity; MET-hrs/wk, metabolic equivalent of task-hours per week.

Table S6 – Physical activity characteristics of colorectal cancer patients at diagnosis who either returned 1 or at least 3 SQUASH questionnaires.

	Surgery only CRC		(Neo-)adjuvant CRC		mCRC	
	1 SQUASH (N=425)	≥3 SQUASH (N=2506)	1 SQUASH (N=275)	≥3 SQUASH (N=1810)	1 SQUASH (N=276)	≥3 SQUASH (N=257)
**Sex**						
Male	245 (58 %)	1512 (60 %)	168 (61 %)	1091 (60 %)	183 (66 %)	153 (60 %)
Female	180 (42 %)	994 (40 %)	107 (39 %)	719 (40 %)	93 (34 %)	104 (40 %)
**Age**						
Median (IQR)	68 (60, 73)	68 (62, 74)	63 (56, 71)	64 (57, 70)	65 (55, 72)	63 (55, 70)
**Recreational PA (MET-hrs/wk)**						
Median (IQR)	21 (5, 42)	29 (12, 51)	21 (7, 42)	26 (12, 47)	20 (5, 43)	27 (11, 45)
**Total PA (MET-hrs/wk)**						
Median (IQR)	75 (31, 131)	93 (57, 135)	93 (47, 145)	99 (64, 143)	76 (35, 132)	97 (63, 142)
**Total MVPA (MET-hrs/wk)**						
Median (IQR)	38 (17, 81)	52 (27, 90)	43 (22, 85)	54 (28, 92)	42 (16, 88)	55 (29, 92)
**PA guideline**						
Non-adherence	256 (68 %)	1420 (57 %)	163 (64 %)	1016 (56 %)	176 (65 %)	153 (60 %)
Adherence	118 (32 %)	1080 (43 %)	92 (36 %)	789 (44 %)	95 (35 %)	103 (40 %)

Abbreviations: CRC, colorectal cancer; FU, FU time in months; IQR, interquartile range; HR, hazard ratio; CI, confidence interval; Ref., reference; PA, physical activity; MET-hrs/wk, metabolic equivalent of task-hours per week; MVPA, moderate and vigorous physical activity.

Table S7 – Timepoint sensitivity analyses of Cox proportional hazard models for overall survival, with missing recreational physical activity (MET-hours/week) revalued as low or high.”

		Surgery only CRC			(Neo-)adjuvantly treated CRC			mCRC		
**Time**	**Category**	**FU (IQR)**	**Death/total**	**HR (95% CI)**	**FU (IQR)**	**Death/total**	**HR (95% CI)**	**FU (IQR)**	**Death/total**	**HR (95% CI)**
T0 (NA = NA)	Low	48 (28-72)	113/986	Ref.	48 (30-75)	143/716	Ref.	20 (11-33)	173/237	Ref.
T0 (NA = Low)	Low	47 (28-71)	121/1049	Ref.	48 (29-75)	148/747	Ref.	21 (11-33)	177/244	Ref.
T0 (NA = Moderate/high)	Low	48 (28-72)	113/986	Ref.	48 (30-75)	143/716	Ref.	20 (11-33)	173/237	Ref.
T0 (NA = NA)	Moderate/high	42 (26-69)	159/2346	**0.663 (0.520 - 0.845)**	46 (27-69)	230/1658	**0.738 (0.599 - 0.909)**	27 (16-42)	268/423	**0.698 (0.575 - 0.848)**
T0 (NA = Low)	Moderate/high	42 (26-69)	159/2346	**0.646 (0.509 - 0.820)**	46 (27-69)	230/1658	**0.739 (0.601 - 0.909)**	27 (16-42)	268/423	**0.712 (0.587 - 0.865)**
T0 (NA = Moderate/high)	Moderate/high	42 (26-68)	167/2409	**0.682 (0.536 - 0.868)**	45 (27-69)	235/1689	**0.741 (0.602 - 0.912)**	27 (16-42)	272/430	**0.692 (0.570 - 0.841)**
T6 (NA = NA)	Low	50 (30-79)	101/814	Ref.	56 (33-84)	115/667	Ref.	33 (21-55)	99/152	Ref.
T6 (NA = Low)	Low	43 (27-68)	147/1376	Ref.	48 (27-74)	183/1050	Ref.	25 (16-38)	214/367	Ref.
T6 (NA = Moderate/high)	Low	50 (30-79)	101/814	Ref.	56 (33-84)	115/667	Ref.	33 (21-55)	99/152	Ref.
T6 (NA = NA)	Moderate/high	43 (28-71)	110/1996	**0.529 (0.403 - 0.696)**	47 (28-69)	172/1332	0.895 (0.705 - 1.137)	32 (20-46)	149/218	1.197 (0.919 - 1.559)
T6 (NA = Low)	Moderate/high	43 (28-71)	110/1996	**0.539 (0.420 - 0.693)**	47 (28-69)	172/1332	**0.774 (0.628 - 0.955)**	32 (20-46)	149/218	1.009 (0.815 - 1.251)
T6 (NA = Moderate/high)	Moderate/high	42 (27-67)	156/2558	**0.630 (0.488 - 0.813)**	43 (27-67)	240/1715	1.004 (0.802 - 1.258)	26 (16-40)	264/433	1.230 (0.968 - 1.564)
T12 (NA = NA)	Low	37 (25-54)	38/501	Ref.	47 (30-70)	76/428	Ref.	31 (23-44)	61/82	Ref.
T12 (NA = Low)	Low	58 (32-93)	206/1909	Ref.	49 (30-77)	204/1087	Ref.	28 (21-41)	192/330	Ref.
T12 (NA = Moderate/high)	Low	37 (25-54)	38/501	Ref.	47 (30-70)	76/428	Ref.	31 (23-44)	61/82	Ref.
T12 (NA = NA)	Moderate/high	36 (24-53)	40/1425	**0.416 (0.264 - 0.656)**	48 (29-69)	115/1240	**0.538 (0.402 - 0.719)**	39 (30-55)	83/155	**0.569 (0.399 - 0.811)**
T12 (NA = Low)	Moderate/high	36 (24-53)	40/1425	**0.388 (0.266 - 0.567)**	48 (29-69)	115/1240	**0.528 (0.416 - 0.670)**	39 (30-55)	83/155	**0.590 (0.451 - 0.771)**
T12 (NA = Moderate/high)	Moderate/high	48 (28-74)	208/2833	**0.623 (0.426 - 0.913)**	49 (29-72)	243/1899	**0.711 (0.549 - 0.922)**	32 (23-48)	214/403	**0.739 (0.548 - 0.996)**
T24 (NA = NA)	Low	61 (39-91)	58/518	Ref.	63 (39-89)	66/369	Ref.	46 (34-67)	34/50	Ref.
T24 (NA = Low)	Low	53 (37-72)	132/1295	Ref.	53 (37-75)	145/907	Ref.	38 (32-57)	99/202	Ref.
T24 (NA = Moderate/high)	Low	61 (39-91)	58/518	Ref.	63 (39-89)	66/369	Ref.	46 (34-67)	34/50	Ref.
T24 (NA = NA)	Moderate/high	57 (38-86)	68/1355	**0.497 (0.348 - 0.709)**	58 (40-82)	92/992	**0.557 (0.405 - 0.767)**	48 (37-67)	49/99	0.712 (0.443 - 1.143)
T24 (NA = Low)	Moderate/high	57 (38-86)	68/1355	**0.404 (0.301 - 0.544)**	58 (40-82)	92/992	**0.515 (0.396 - 0.670)**	48 (37-67)	49/99	0.767 (0.540 - 1.088)
T24 (NA = Moderate/high)	Moderate/high	53 (37-75)	142/2132	0.737 (0.541 - 1.005)	55 (38-77)	171/1530	**0.740 (0.555 - 0.986)**	41 (33-59)	114/251	0.862 (0.568 - 1.307)

PA categorization for each timepoint was based on was based on cut-offs from tertiles created in an age- and sex-matched sample of the general population. Low: tertile 1, moderate: tertile 2, high: tertile 3. Missing PA information from all patients with FU time > 3 months than the assessed questionnaire was recoded as either high or low activity.
Abbreviations: CRC, colorectal cancer; FU, FU time in months; IQR, interquartile range; HR, hazard ratio; CI, confidence interval; Ref., reference; PA, physical activity; MET-hrs/wk, metabolic equivalent of task-hours per week; NA, missing.

Table S8 – Changes between subsequent timepoint sensitivity analyses of Cox proportional hazard models for overall survival, with missing recreational physical activity (MET-hours/week) revalued as low or high.

		Surgery only CRC			(Neo-)adjuvantly treated CRC			mCRC		
**Time**	**Category**	**FU (IQR)**	**Death/total**	**HR (95% CI)**	**FU (IQR)**	**Death/total**	**HR (95% CI)**	**FU (IQR)**	**Death/total**	**HR (95% CI)**
T0 to T6 (NA = NA)	Remains inactive	50 (29-79)	65/483	Ref.	56 (33-85)	62/348	Ref.	29 (19-41)	57/81	Ref.
T0 to T6 (NA = Low)	Remains inactive	46 (28-71)	94/722	Ref.	49 (29-75)	102/500	Ref.	24 (16-35)	101/157	Ref.
T0 to T6 (NA = Moderate/high)	Remains inactive	50 (29-79)	65/483	Ref.	56 (33-85)	62/348	Ref.	29 (19-41)	57/81	Ref.
T0 to T6 (NA = NA)	Change to inactive	51 (30-80)	34/316	**0.569 (0.347 - 0.934)**	56 (33-83)	52/313	1.045 (0.692 - 1.578)	41 (26-65)	41/69	1.020 (0.638 - 1.629)
T0 to T6 (NA = Low)	Change to inactive	42 (27-65)	53/654	**0.551 (0.346 - 0.879)**	46 (25-71)	81/550	0.820 (0.565 - 1.192)	25 (16-41)	113/210	0.922 (0.603 - 1.410)
T0 to T6 (NA = Moderate/high)	Change to inactive	51 (30-78)	36/331	0.776 (0.527 - 1.143)	56 (33-83)	53/319	1.367 (0.973 - 1.920)	39 (26-64)	42/71	1.224 (0.858 - 1.748)
T0 to T6 (NA = NA)	Change to active	49 (31-74)	21/300	0.747 (0.493 - 1.132)	48 (33-74)	36/223	0.907 (0.627 - 1.312)	30 (18-51)	28/39	**0.609 (0.396 - 0.936)**
T0 to T6 (NA = Low)	Change to active	48 (30-72)	22/322	**0.691 (0.493 - 0.969)**	48 (32-73)	38/239	0.767 (0.573 - 1.027)	30 (18-50)	29/40	**0.724 (0.549 - 0.955)**
T0 to T6 (NA = Moderate/high)	Change to active	45 (28-68)	44/499	0.772 (0.513 - 1.161)	43 (28-68)	73/360	0.910 (0.631 - 1.313)	22 (15-32)	69/109	**0.618 (0.408 - 0.937)**
T0 to T6 (NA = NA)	Remains active	43 (27-71)	88/1674	**0.460 (0.332 - 0.637)**	46 (28-69)	134/1093	0.817 (0.604 - 1.107)	33 (22-45)	120/178	0.922 (0.667 - 1.275)
T0 to T6 (NA = Low)	Remains active	43 (27-71)	88/1674	**0.445 (0.332 - 0.598)**	46 (28-69)	134/1093	**0.652 (0.503 - 0.844)**	33 (22-45)	120/178	0.826 (0.630 - 1.082)
T0 to T6 (NA = Moderate/high)	Remains active	41 (26-67)	112/2059	**0.515 (0.378 - 0.703)**	43 (27-67)	167/1355	0.849 (0.633 - 1.138)	27 (18-41)	195/324	0.924 (0.685 - 1.247)
T6 to T12 (NA = NA)	Remains inactive	37 (26-55)	25/279	Ref.	46 (32-73)	40/243	Ref.	31 (23-41)	33/45	Ref.
T6 to T12 (NA = Low)	Remains inactive	49 (28-73)	125/1058	Ref.	48 (29-75)	133/699	Ref.	27 (21-37)	125/234	Ref.
T6 to T12 (NA = Moderate/high)	Remains inactive	37 (26-55)	25/279	Ref.	46 (32-73)	40/243	Ref.	31 (23-41)	33/45	Ref.
T6 to T12 (NA = NA)	Change to inactive	38 (25-51)	9/170	0.660 (0.320 - 1.361)	48 (28-68)	27/145	**0.529 (0.314 - 0.891)**	31 (20-44)	26/34	**0.499 (0.270 - 0.921)**
T6 to T12 (NA = Low)	Change to inactive	72 (40-104)	81/851	**0.468 (0.259 - 0.845)**	51 (32-81)	71/388	**0.473 (0.316 - 0.709)**	34 (24-46)	67/96	**0.429 (0.277 - 0.665)**
T6 to T12 (NA = Moderate/high)	Change to inactive	39 (25-54)	13/222	0.712 (0.430 - 1.179)	47 (27-67)	36/185	0.801 (0.537 - 1.195)	31 (20-44)	28/37	0.657 (0.410 - 1.052)
T6 to T12 (NA = NA)	Change to active	41 (27-54)	11/183	0.623 (0.288 - 1.347)	58 (35-82)	22/230	1.271 (0.778 - 2.076)	44 (35-66)	19/44	1.459 (0.841 - 2.533)
T6 to T12 (NA = Low)	Change to active	39 (25-55)	13/293	**0.666 (0.497 - 0.894)**	52 (29-74)	29/316	0.934 (0.699 - 1.249)	43 (35-60)	25/58	1.187 (0.872 - 1.614)
T6 to T12 (NA = Moderate/high)	Change to active	63 (36-98)	71/528	0.652 (0.333 - 1.279)	64 (35-90)	65/412	1.358 (0.864 - 2.134)	41 (28-62)	47/87	1.303 (0.778 - 2.184)
T6 to T12 (NA = NA)	Remains active	35 (24-52)	27/1132	**0.302 (0.172 - 0.529)**	46 (28-67)	86/924	**0.633 (0.433 - 0.925)**	36 (27-50)	57/96	0.831 (0.519 - 1.331)
T6 to T12 (NA = Low)	Remains active	35 (24-52)	27/1132	**0.309 (0.199 - 0.481)**	46 (28-67)	86/924	**0.533 (0.403 - 0.704)**	36 (27-50)	57/96	0.756 (0.547 - 1.044)
T6 to T12 (NA = Moderate/high)	Remains active	43 (28-70)	137/2305	**0.485 (0.307 - 0.765)**	44 (28-68)	178/1487	0.817 (0.579 - 1.153)	30 (22-43)	166/315	0.883 (0.597 - 1.306)
T12 to T24 (NA = NA)	Remains inactive	46 (34-59)	13/168	Ref.	56 (38-78)	27/144	Ref.	47 (33-62)	15/21	Ref.
T12 to T24 (NA = Low)	Remains inactive	57 (38-81)	116/990	Ref.	56 (37-79)	115/613	Ref.	38 (31-58)	69/144	Ref.
T12 to T24 (NA = Moderate/high)	Remains inactive	46 (34-59)	13/168	Ref.	56 (38-78)	27/144	Ref.	47 (33-62)	15/21	Ref.
T12 to T24 (NA = NA)	Change to inactive	41 (33-59)	3/113	NA	56 (38-81)	14/124	0.609 (0.319 - 1.162)	48 (42-62)	7/13	NA
T12 to T24 (NA = Low)	Change to inactive	41 (34-58)	16/305	**0.469 (0.332 - 0.664)**	50 (37-69)	30/294	**0.583 (0.402 - 0.845)**	40 (34-52)	30/58	1.159 (0.689 - 1.950)
T12 to T24 (NA = Moderate/high)	Change to inactive	72 (48-104)	45/350	0.934 (0.438 - 1.993)	66 (40-93)	39/225	0.760 (0.450 - 1.284)	45 (34-71)	19/29	1.293 (0.593 - 2.820)
T12 to T24 (NA = NA)	Change to active	43 (35-59)	4/115	NA	55 (39-76)	14/119	0.603 (0.316 - 1.154)	45 (35-71)	6/8	NA
T12 to T24 (NA = Low)	Change to active	89 (61-112)	53/607	0.815 (0.465 - 1.428)	67 (44-103)	38/268	0.671 (0.445 - 1.012)	45 (37-76)	21/33	0.966 (0.619 - 1.507)
T12 to T24 (NA = Moderate/high)	Change to active	43 (34-57)	14/209	0.728 (0.370 - 1.432)	52 (38-73)	29/210	0.706 (0.429 - 1.163)	39 (31-48)	14/28	1.154 (0.542 - 2.456)
T12 to T24 (NA = NA)	Remains active	43 (34-59)	15/748	NA	57 (38-77)	54/723	**0.393 (0.247 - 0.625)**	49 (38-63)	28/66	NA

T12 to T24 (NA = Low)	Remains active	43 (34-59)	15/748	**0.265 (0.149 - 0.471)**	57 (38-77)	54/723	**0.409 (0.294 - 0.570)**	49 (38-63)	28/66	**0.598 (0.377 - 0.947)**
T12 to T24 (NA = Moderate/high)	Remains active	55 (38-79)	128/1923	0.556 (0.303 - 1.020)	56 (38-77)	142/1319	**0.573 (0.379 - 0.865)**	41 (33-60)	100/223	0.901 (0.491 - 1.654)

PA categorization was based on cut-offs from tertiles created in an age- and sex-matched sample of the general population. Low: tertile 1, moderate: tertile 2, high: tertile 3. Missing PA information from all patients with FU time > 3 months than the assessed questionnaire was recoded as either high or low activity. Remains inactive: low at both timepoints; change to inactive: moderate/high at first- and low at second timepoint; change to active: low at first- and moderate/high at second timepoint; remains active: tertile 2/3 at both timepoints.
Abbreviations: CRC, colorectal cancer; FU, FU time in months; IQR, interquartile range; HR, hazard ratio; CI, confidence interval; Ref., reference; PA, physical activity; MET-hrs/wk, metabolic equivalent of task-hours per week; NA, missing.

Table S9 – Timepoint sensitivity analyses of Cox proportional hazard models for recreational physical activity and overall survival, with varying covariate adjustments and follow-up restrictions.

		Surgery only CRC			(Neo-)adjuvantly treated CRC			mCRC		
**Time, analysis**	**Category**	**FU (IQR)**	**Death/total**	**HR (95% CI)**	**FU (IQR)**	**Death/total**	**HR (95% CI)**	**FU (IQR)**	**Death/total**	**HR (95% CI)**
T0, Unadjusted	Low	48 (28-72)	113/986	Ref.	48 (30-75)	143/716	Ref.	20 (11-33)	174/238	Ref.
T0, Multivariable	Low	48 (28-72)	109/975	Ref.	48 (30-75)	139/706	Ref.	19 (11-32)	163/225	Ref.
T0, Mult. (≥6 mo FU)	Low	48 (29-72)	105/971	Ref.	49 (30-76)	134/701	Ref.	22 (14-34)	138/200	Ref.
T0, Mult.+sociodemographics	Low	48 (28-72)	108/967	Ref.	49 (30-76)	134/696	Ref.	19 (11-32)	161/223	Ref.
T0, Mult. + PF	Low	48 (28-72)	108/969	Ref.	48 (29-75)	137/697	Ref.	19 (11-32)	162/224	Ref.
T0, Unadjusted	Moderate	44 (28-72)	92/1203	**0.670 (0.509 - 0.882)**	46 (27-71)	131/910	**0.774 (0.610 - 0.981)**	27 (16-42)	150/237	**0.660 (0.530 - 0.822)**
T0, Multivariable	Moderate	44 (28-72)	90/1186	**0.718 (0.542 - 0.952)**	46 (27-70)	128/898	**0.782 (0.614 - 0.996)**	28 (17-43)	138/222	**0.645 (0.511 - 0.813)**
T0, Mult. (≥6 mo FU)	Moderate	44 (28-72)	83/1179	**0.683 (0.511 - 0.912)**	46 (27-70)	123/893	**0.776 (0.607 - 0.994)**	29 (19-46)	128/212	**0.666 (0.521 - 0.852)**
T0, Mult.+sociodemographics	Moderate	44 (28-72)	90/1184	0.787 (0.592 - 1.046)	46 (27-70)	127/894	0.808 (0.631 - 1.035)	28 (17-43)	138/222	**0.629 (0.495 - 0.800)**
T0, Mult. + PF	Moderate	44 (28-71)	90/1182	0.810 (0.609 - 1.077)*	46 (27-70)	126/895	0.794 (0.620 - 1.015)	28 (17-44)	137/221	**0.678 (0.536 - 0.858)**
T0, Unadjusted	High	40 (25-65)	67/1143	**0.580 (0.429 - 0.785)**	45 (28-68)	99/748	**0.726 (0.562 - 0.938)**	26 (15-42)	118/187	**0.707 (0.560 - 0.894)**
T0, Multivariable	High	40 (25-65)	66/1117	**0.615 (0.451 - 0.838)**	44 (28-68)	97/739	**0.706 (0.543 - 0.917)**	26 (15-42)	110/177	**0.691 (0.538 - 0.888)**
T0, Mult. (≥6 mo FU)	High	40 (25-65)	59/1110	**0.570 (0.413 - 0.787)**	45 (28-68)	96/738	**0.721 (0.553 - 0.939)**	27 (16-43)	101/168	**0.707 (0.542 - 0.923)**
T0, Mult.+sociodemographics	High	40 (25-65)	65/1109	**0.654 (0.479 - 0.893)**	45 (28-68)	96/737	**0.725 (0.555 - 0.947)**	26 (15-42)	110/177	**0.692 (0.535 - 0.896)**
T0, Mult. + PF	High	40 (25-64)	66/1114	**0.718 (0.524 - 0.985)***	44 (28-68)	97/738	**0.731 (0.561 - 0.954)**	26 (15-42)	110/177	**0.729 (0.565 - 0.940)**
T0, Unadjusted	Continuous	43 (27-70)	272/3332	**0.691 (0.574 - 0.871)**	47 (28-71)	373/2374	**0.794 (0.691 - 0.955)**	24 (14-40)	442/662	**0.794 (0.691 - 0.955)**
T0, Multivariable	Continuous	43 (27-69)	265/3278	**0.724 (0.601 - 0.912)**	46 (28-71)	364/2343	**0.794 (0.660 - 0.912)**	24 (14-40)	411/624	**0.794 (0.691 - 0.955)**
T0, Mult. (≥6 mo FU)	Continuous	43 (27-69)	247/3260	**0.724 (0.574 - 0.871)**	47 (28-71)	353/2332	**0.794 (0.660 - 0.912)**	26 (16-41)	367/580	**0.832 (0.691 - 1.000)**
T0, Mult.+sociodemographics	Continuous	43 (27-69)	263/3260	**0.758 (0.630 - 0.912)**	46 (28-71)	357/2327	**0.794 (0.660 - 0.955)**	24 (14-40)	409/622	**0.832 (0.691 - 0.955)**
T0, Mult. + PF	Continuous	43 (27-69)	264/3265	**0.794 (0.660 - 1.000)**	46 (28-71)	360/2330	**0.794 (0.660 - 0.955)**	24 (14-40)	409/622	**0.832 (0.691 - 1.000)**
T6, Unadjusted	Low	50 (30-79)	101/814	Ref.	56 (33-84)	115/667	Ref.	33 (21-55)	99/152	Ref.
T6, Multivariable	Low	52 (30-81)	96/775	Ref.	57 (33-85)	112/647	Ref.	34 (21-57)	95/146	Ref.
T6, Mult. (≥6 mo FU)	Low	52 (31-81)	93/772	Ref.	58 (34-85)	107/642	Ref.	35 (24-59)	83/134	Ref.
T6, Mult.+sociodemographics	Low	52 (30-81)	95/769	Ref.	57 (33-85)	110/641	Ref.	34 (21-57)	95/146	Ref.
T6, Mult. + PF	Low	51 (30-80)	95/771	Ref.	57 (33-84)	111/640	Ref.	34 (21-56)	95/145	Ref.
T6, Unadjusted	Moderate	47 (28-74)	67/1080	**0.537 (0.395 - 0.732)**	50 (30-77)	114/811	0.896 (0.691 - 1.161)	31 (19-46)	102/145	1.197 (0.908 - 1.580)*
T6, Multivariable	Moderate	48 (28-74)	65/1050	**0.585 (0.426 - 0.804)**	50 (31-78)	112/798	0.934 (0.717 - 1.216)	31 (19-47)	100/142	**1.351 (1.006 - 1.816)**
T6, Mult. (≥6 mo FU)	Moderate	48 (28-74)	65/1050	**0.604 (0.438 - 0.831)**	51 (31-78)	106/792	0.931 (0.710 - 1.220)	34 (22-48)	89/131	**1.414 (1.031 - 1.939)**
T6, Mult.+sociodemographics	Moderate	48 (28-74)	64/1048	**0.619 (0.449 - 0.854)**	51 (31-78)	109/791	0.933 (0.712 - 1.221)	31 (19-47)	100/142	**1.356 (1.003 - 1.833)**
T6, Mult. + PF	Moderate	48 (28-74)	65/1044	**0.689 (0.497 - 0.953)**	50 (31-78)	112/796	0.949 (0.723 - 1.247)	31 (19-48)	98/140	1.346 (0.999 - 1.812)
T6, Unadjusted	High	41 (26-67)	43/916	**0.450 (0.315 - 0.643)**	41 (28-62)	58/521	0.861 (0.627 - 1.183)	34 (24-45)	47/74	0.978 (0.691 - 1.385)
T6, Multivariable	High	41 (27-67)	43/889	**0.469 (0.325 - 0.676)**	41 (28-62)	57/514	0.893 (0.642 - 1.242)	34 (24-46)	42/68	0.941 (0.647 - 1.370)
T6, Mult. (≥6 mo FU)	High	41 (27-67)	42/888	**0.473 (0.326 - 0.685)**	41 (28-62)	57/513	0.953 (0.683 - 1.330)	34 (25-48)	40/66	1.036 (0.701 - 1.530)*
T6, Mult.+sociodemographics	High	42 (26-68)	43/884	**0.496 (0.343 - 0.717)**	41 (28-62)	56/511	0.903 (0.645 - 1.264)	34 (24-46)	42/68	0.934 (0.638 - 1.369)
T6, Mult. + PF	High	41 (26-67)	42/879	**0.574 (0.393 - 0.839)***	41 (28-62)	57/512	0.912 (0.652 - 1.276)	34 (24-46)	42/68	0.978 (0.669 - 1.428)

T6, Unadjusted	Continuous	45 (28-73)	211/2810	**0.574 (0.434 - 0.758)**	50 (30-75)	287/1999	0.955 (0.794 - 1.148)	33 (21-48)	248/371	0.955 (0.794 - 1.148)
T6, Multivariable	Continuous	47 (28-74)	204/2714	**0.574 (0.454 - 0.758)**	50 (30-76)	281/1959	0.955 (0.794 - 1.202)	33 (21-49)	237/356	0.955 (0.794 - 1.202)
T6, Mult. (≥6 mo FU)	Continuous	47 (28-74)	200/2710	**0.601 (0.454 - 0.758)**	50 (31-76)	270/1947	1.000 (0.832 - 1.258)	34 (24-51)	212/331	1.047 (0.832 - 1.258)
T6, Mult.+sociodemographics	Continuous	47 (28-74)	202/2701	**0.601 (0.476 - 0.794)**	50 (30-76)	275/1943	1.000 (0.794 - 1.202)	33 (21-49)	237/356	0.955 (0.758 - 1.148)
T6, Mult. + PF	Continuous	46 (28-74)	202/2694	**0.691 (0.523 - 0.912)***	50 (30-75)	280/1948	1.000 (0.832 - 1.202)	33 (21-49)	235/353	1.000 (0.832 - 1.202)
T12, Unadjusted	Low	37 (25-54)	38/501	Ref.	47 (30-70)	76/428	Ref.	31 (23-44)	61/82	Ref.
T12, Multivariable	Low	38 (26-55)	37/455	Ref.	48 (31-70)	72/405	Ref.	31 (22-44)	58/79	Ref.
T12, Mult. (≥6 mo FU)	Low	41 (30-57)	34/407	Ref.	50 (34-72)	68/382	Ref.	33 (26-47)	47/68	Ref.
T12, Mult.+sociodemographics	Low	38 (26-55)	35/450	Ref.	48 (32-70)	70/402	Ref.	31 (22-44)	58/79	Ref.
T12, Mult. + PF	Low	38 (26-55)	37/454	Ref.	48 (32-70)	71/402	Ref.	31 (22-44)	58/79	Ref.
T12, Unadjusted	Moderate	37 (24-53)	24/730	**0.451 (0.271 - 0.752)**	49 (29-73)	64/688	**0.514 (0.369 - 0.717)**	39 (30-53)	57/96	**0.602 (0.419 - 0.865)**
T12, Multivariable	Moderate	37 (24-53)	23/699	**0.461 (0.271 - 0.782)**	50 (29-74)	64/669	**0.536 (0.382 - 0.753)**	39 (30-56)	53/92	**0.563 (0.378 - 0.840)**
T12, Mult. (≥6 mo FU)	Moderate	40 (28-55)	22/615	**0.485 (0.281 - 0.836)**	52 (33-76)	55/617	**0.483 (0.338 - 0.691)**	41 (32-59)	48/87	**0.589 (0.381 - 0.910)**
T12, Mult.+sociodemographics	Moderate	37 (24-53)	22/694	**0.509 (0.295 - 0.880)***	50 (29-74)	63/664	**0.529 (0.374 - 0.748)**	39 (30-56)	53/92	**0.547 (0.365 - 0.820)**
T12, Mult. + PF	Moderate	37 (24-53)	23/699	**0.528 (0.307 - 0.907)***	50 (29-74)	63/667	**0.590 (0.417 - 0.834)***	39 (31-55)	52/90	0.668 (0.437 - 1.020)*
T12, Unadjusted	High	35 (24-53)	16/695	**0.324 (0.181 - 0.582)**	46 (28-65)	51/552	**0.568 (0.398 - 0.810)**	39 (31-56)	26/59	**0.440 (0.278 - 0.697)***
T12, Multivariable	High	35 (24-53)	16/670	**0.350 (0.191 - 0.640)**	47 (29-65)	49/538	**0.549 (0.379 - 0.794)**	39 (31-56)	26/59	**0.489 (0.301 - 0.794)**
T12, Mult. (≥6 mo FU)	High	38 (28-55)	15/605	**0.361 (0.193 - 0.675)**	51 (33-67)	44/489	**0.524 (0.356 - 0.771)**	39 (31-56)	26/59	**0.581 (0.349 - 0.967)***
T12, Mult.+sociodemographics*	High	35 (24-53)	16/667	**0.407 (0.221 - 0.751)**	47 (29-65)	47/533	**0.527 (0.360 - 0.772)**	39 (31-56)	26/59	**0.498 (0.303 - 0.818)**
T12, Mult. + PF*	High	35 (24-53)	16/668	**0.461 (0.246 - 0.866)**	47 (29-65)	48/537	**0.624 (0.426 - 0.914)***	38 (31-54)	26/58	0.625 (0.380 - 1.030)*
T12, Unadjusted	Continuous	37 (24-53)	78/1926	**0.359 (0.214 - 0.574)**	48 (29-69)	191/1668	**0.758 (0.574 - 0.955)**	36 (26-51)	144/237	**0.601 (0.414 - 0.832)**
T12, Multivariable	Continuous	37 (24-54)	76/1824	**0.377 (0.224 - 0.630)**	48 (29-69)	185/1612	**0.724 (0.574 - 0.955)**	36 (26-52)	137/230	**0.660 (0.454 - 0.955)**
T12, Mult. (≥6 mo FU)	Continuous	39 (29-55)	71/1627	**0.414 (0.246 - 0.660)**	51 (34-72)	167/1488	**0.724 (0.574 - 0.955)**	38 (29-54)	121/214	0.724 (0.499 - 1.047)
T12, Mult.+sociodemographics	Continuous	37 (24-54)	73/1811	**0.434 (0.271 - 0.724)***	49 (29-70)	180/1599	**0.724 (0.574 - 0.955)**	36 (26-52)	137/230	**0.660 (0.454 - 0.955)**
T12, Mult. + PF	Continuous	37 (24-54)	76/1821	**0.454 (0.284 - 0.758)***	48 (29-69)	182/1606	0.794 (0.630 - 1.000)	36 (26-51)	136/227	0.758 (0.523 - 1.096)*
T24, Unadjusted	Low	61 (39-91)	58/518	Ref.	63 (39-89)	66/369	Ref.	46 (34-67)	34/50	Ref.
T24, Multivariable	Low	62 (40-95)	55/477	Ref.	63 (40-89)	63/350	Ref.	46 (34-67)	34/50	Ref.
T24, Mult. (≥6 mo FU)	Low	65 (48-98)	53/428	Ref.	66 (47-92)	55/319	Ref.	49 (37-71)	28/44	Ref.
T24, Mult.+sociodemographics	Low	62 (40-95)	54/475	Ref.	63 (40-89)	63/350	Ref.	46 (34-67)	34/50	Ref.
T24, Mult. + PF	Low	62 (39-93)	53/470	Ref.	63 (39-89)	62/345	Ref.	47 (34-67)	33/49	Ref.
T24, Unadjusted	Moderate	59 (39-88)	39/695	**0.494 (0.329 - 0.742)**	60 (40-85)	60/568	**0.608 (0.429 - 0.863)***	47 (36-66)	33/58	0.785 (0.486 - 1.269)
T24, Multivariable	Moderate	60 (40-90)	38/662	**0.535 (0.352 - 0.813)**	60 (40-85)	59/549	**0.682 (0.476 - 0.978)**	46 (36-65)	33/57	0.765 (0.454 - 1.288)
T24, Mult. (≥6 mo FU)	Moderate	64 (46-94)	37/596	**0.535 (0.352 - 0.813)**	64 (47-88)	58/491	**0.696 (0.485 - 0.999)**	48 (37-66)	30/53	0.765 (0.454 - 1.288)
T24, Mult.+sociodemographics	Moderate	60 (40-90)	38/661	**0.578 (0.379 - 0.883)**	60 (40-85)	59/546	0.698 (0.486 - 1.003)	46 (36-65)	33/57	0.707 (0.393 - 1.272)
T24, Mult. + PF	Moderate	60 (40-89)	37/655	**0.648 (0.420 - 0.999)***	60 (40-84)	55/541	**0.681 (0.469 - 0.990)**	46 (36-63)	32/56	1.155 (0.642 - 2.077)*
T24, Unadjusted	High	55 (37-83)	29/660	**0.428 (0.274 - 0.669)**	57 (40-80)	32/424	**0.469 (0.308 - 0.717)**	52 (42-68)	16/41	**0.480 (0.265 - 0.870)**
T24, Multivariable	High	55 (37-83)	27/616	**0.446 (0.279 - 0.712)**	57 (40-79)	31/407	**0.510 (0.329 - 0.791)**	53 (42-68)	15/40	**0.487 (0.253 - 0.939)**
T24, Mult. (≥6 mo FU)	High	60 (42-88)	25/550	**0.446 (0.279 - 0.712)**	60 (46-81)	30/370	**0.521 (0.336 - 0.809)**	54 (42-69)	14/39	**0.487 (0.253 - 0.939)**

T24, Mult.+sociodemographics	High	55 (37-83)	27/611	**0.490 (0.306 - 0.786)**	57 (40-79)	31/405	**0.523 (0.337 - 0.813)**	53 (42-68)	15/40	**0.480 (0.232 - 0.994)**
T24, Mult. + PF	High	55 (37-82)	27/609	**0.599 (0.368 - 0.976)***	57 (40-79)	31/406	**0.537 (0.344 - 0.839)**	53 (42-68)	15/40	**0.875 (0.433 - 1.769)***
T24, Unadjusted	Continuous	58 (38-87)	126/1873	**0.574 (0.395 - 0.794)**	60 (40-84)	158/1361	**0.630 (0.476 - 0.871)**	48 (36-67)	83/149	**0.548 (0.359 - 0.794)**
T24, Multivariable	Continuous	59 (39-88)	120/1755	**0.601 (0.414 - 0.832)**	60 (40-84)	153/1306	**0.660 (0.499 - 0.912)**	48 (36-67)	82/147	**0.548 (0.343 - 0.832)**
T24, Mult. (≥6 mo FU)	Continuous	63 (44-93)	115/1574	**0.601 (0.414 - 0.832)**	63 (46-87)	143/1180	**0.691 (0.499 - 0.912)**	50 (38-68)	72/136	**0.548 (0.343 - 0.832)**
T24, Mult.+sociodemographics	Continuous	59 (39-88)	119/1747	**0.630 (0.454 - 0.871)**	60 (40-84)	153/1301	**0.691 (0.499 - 0.912)**	48 (36-67)	82/147	**0.523 (0.327 - 0.871)**
T24, Mult. + PF	Continuous	59 (39-87)	117/1734	0.724 (0.499 - 1.000)*	60 (40-84)	148/1292	**0.691 (0.523 - 0.955)**	48 (36-66)	80/145	0.758 (0.476 - 1.148)*

Effect estimates for continuous activity are shown per 1 SD increase (46 MET-hrs/wk).
PA categorization was based on cut-offs from tertiles created in an age- and sex-matched sample of the general population. Low (reference group): tertile 1, moderate: tertile 2, high: tertile 3.
Unadjusted models included only PA as exposure. Mult. (≥6 mo FU): restriction to patients with ≥6 months FU after last used PA questionnaire. Mult. (expanded): further adjustment for smoking status (current, prior, former), education (low, moderate, high), marital status (single, married/in-law). Mult. + PF: further adjustment for functional status using the revised physical functioning (PF2) subscale of the EORTC QLQ-C30 at the time of PA assessment (dichotomized at >66.7 vs. ≤66.7).
*Estimates that changed >10% compared to the multivariable model.
Abbreviations: CRC, colorectal cancer; FU, FU time in months; IQR, interquartile range; HR, hazard ratio; CI, confidence interval; Ref., reference; PA, physical activity; MET-hrs/wk, metabolic equivalent of task-hours per week; EORTC, European Organisation for Research and Treatment of Cancer; QLQ-C30, Quality of Life Questionnaire-Core 30; PF2, PF subscale (second version).

Table S10 – Changes between subsequent timepoint sensitivity analyses of Cox proportional hazard models for recreational physical activity and overall survival, with varying covariate adjustments and follow-up restrictions.

		Surgery only CRC			(Neo-)adjuvantly treated CRC			mCRC		
Time, analysis	Category	FU (IQR)	Death/total	HR (95% CI)	FU (IQR)	Death/total	HR (95% CI)	FU (IQR)	Death/total	HR (95% CI)
T0 to T6, Unadjusted	Remains inactive	50 (29-79)	65/483	Ref.	56 (33-85)	62/348	Ref.	29 (19-41)	57/81	Ref.
T0 to T6, Multivariable	Remains inactive	50 (29-79)	63/477	Ref.	57 (33-86)	59/341	Ref.	29 (18-41)	53/77	Ref.
T0 to T6, Mult. (≥6 mo FU)	Remains inactive	50 (29-79)	60/474	Ref.	57 (33-86)	56/338	Ref.	32 (22-44)	44/68	Ref.
T0 to T6, Mult.+sociodemographics	Remains inactive	50 (29-79)	62/470	Ref.	57 (33-86)	57/336	Ref.	29 (18-41)	53/77	Ref.
T0 to T6, Mult. + PF	Remains inactive	50 (29-79)	63/475	Ref.	55 (33-85)	57/336	Ref.	29 (18-41)	53/77	Ref.
T0 to T6, Unadjusted	Changes to inactive	49 (31-74)	21/300	0.762 (0.503 - 1.154)	48 (33-74)	36/223	0.946 (0.654 - 1.368)	30 (18-51)	28/39	**0.586 (0.391 - 0.877)**
T0 to T6, Multivariable	Changes to inactive	49 (30-74)	21/299	0.746 (0.491 - 1.134)	48 (33-73)	36/222	0.933 (0.640 - 1.358)	27 (17-47)	26/35	**0.605 (0.386 - 0.948)**
T0 to T6, Mult. (≥6 mo FU)	Changes to inactive	49 (30-74)	21/299	0.781 (0.512 - 1.191)	48 (33-73)	36/222	0.943 (0.642 - 1.386)	29 (18-48)	24/33	0.625 (0.385 - 1.014)
T0 to T6, Mult.+sociodemographics	Changes to inactive	49 (30-74)	21/299	0.809 (0.530 - 1.235)	48 (33-75)	34/218	0.953 (0.650 - 1.397)	27 (17-47)	26/35	**0.594 (0.378 - 0.935)**
T0 to T6, Mult. + PF	Changes to inactive	49 (30-74)	20/296	0.824 (0.540 - 1.255)*	48 (33-73)	36/218	0.963 (0.658 - 1.409)	27 (17-47)	26/35	**0.630 (0.401 - 0.988)**
T0 to T6, Unadjusted	Changes to active	51 (30-80)	34/316	**0.523 (0.320 - 0.855)***	56 (33-83)	52/313	1.006 (0.667 - 1.518)	41 (26-65)	41/69	0.876 (0.556 - 1.378)*
T0 to T6, Multivariable	Changes to active	51 (30-76)	34/312	**0.582 (0.354 - 0.956)**	56 (33-83)	51/311	1.092 (0.720 - 1.656)	42 (25-65)	39/66	1.183 (0.730 - 1.917)
T0 to T6, Mult. (≥6 mo FU)	Changes to active	51 (30-76)	34/312	0.607 (0.368 - 1.000)	56 (33-83)	49/309	1.155 (0.758 - 1.758)	43 (27-67)	35/62	1.325 (0.792 - 2.217)*
T0 to T6, Mult.+sociodemographics	Changes to active	51 (30-76)	34/312	**0.600 (0.365 - 0.987)**	56 (33-83)	51/310	1.065 (0.694 - 1.636)	42 (25-65)	39/66	1.132 (0.694 - 1.846)
T0 to T6, Mult. + PF	Changes to active	51 (30-76)	34/311	0.627 (0.377 - 1.045)	56 (33-83)	51/311	1.141 (0.750 - 1.737)	42 (25-65)	39/66	1.182 (0.729 - 1.917)
T0 to T6, Unadjusted	Remains active	43 (27-71)	88/1674	**0.439 (0.318 - 0.605)**	46 (28-69)	134/1093	0.829 (0.613 - 1.121)	33 (21-45)	120/179	0.855 (0.623 - 1.172)
T0 to T6, Multivariable	Remains active	42 (26-70)	87/1644	**0.462 (0.332 - 0.643)**	46 (28-68)	131/1077	0.855 (0.625 - 1.170)	34 (22-47)	109/167	0.855 (0.610 - 1.200)
T0 to T6, Mult. (≥6 mo FU)	Remains active	42 (27-70)	86/1643	**0.479 (0.343 - 0.670)**	46 (28-68)	126/1071	0.872 (0.633 - 1.202)	34 (24-48)	101/159	0.944 (0.655 - 1.361)*
T0 to T6, Mult.+sociodemographics	Remains active	42 (26-70)	87/1639	**0.510 (0.366 - 0.712)***	46 (28-68)	129/1072	0.864 (0.625 - 1.194)	34 (22-47)	109/167	0.859 (0.601 - 1.228)
T0 to T6, Mult. + PF	Remains active	42 (26-69)	87/1639	**0.522 (0.373 - 0.731)***	46 (28-68)	129/1074	0.885 (0.641 - 1.222)	34 (22-48)	108/166	0.896 (0.635 - 1.263)
T0 to T6, Unadjusted	Continuous	45 (28-73)	208/2773	0.912 (0.758 - 1.096)	50 (30-75)	284/1977	1.096 (0.912 - 1.258)	33 (21-48)	246/368	1.047 (0.912 - 1.202)
T0 to T6, Multivariable	Continuous	45 (28-72)	205/2732	0.912 (0.758 - 1.096)	50 (30-75)	277/1951	1.148 (0.955 - 1.317)	33 (21-49)	227/345	1.096 (0.912 - 1.258)
T0 to T6, Mult. (≥6 mo FU)	Continuous	45 (28-72)	201/2728	0.912 (0.758 - 1.096)	50 (30-75)	267/1940	1.202 (1.000 - 1.378)	34 (23-51)	204/322	1.096 (0.955 - 1.317)
T0 to T6, Mult.+sociodemographics	Continuous	45 (28-73)	204/2720	0.912 (0.724 - 1.096)	50 (30-75)	271/1936	1.096 (0.912 - 1.317)	33 (21-49)	227/345	1.047 (0.912 - 1.258)
T0 to T6, Mult. + PF	Continuous	44 (28-72)	204/2721	0.912 (0.758 - 1.096)	49 (30-74)	273/1939	1.096 (0.955 - 1.317)	33 (21-49)	226/344	1.047 (0.912 - 1.258)
T6 to T12, Unadjusted	Remains inactive	37 (26-55)	25/279	Ref.	46 (32-73)	40/243	Ref.	31 (23-41)	33/45	Ref.
T6 to T12, Multivariable	Remains inactive	37 (26-55)	21/255	Ref.	48 (33-74)	39/233	Ref.	32 (23-44)	31/43	Ref.
T6 to T12, Mult. (≥6 mo FU)	Remains inactive	39 (30-57)	21/228	Ref.	49 (34-77)	39/224	Ref.	33 (26-50)	27/39	Ref.
T6 to T12, Mult.+sociodemographics	Remains inactive	37 (26-55)	20/252	Ref.	48 (33-75)	38/231	Ref.	32 (23-44)	31/43	Ref.
T6 to T12, Mult. + PF	Remains inactive	37 (26-55)	21/255	Ref.	48 (33-73)	39/231	Ref.	32 (23-44)	31/43	Ref.
T6 to T12, Unadjusted	Changes to inactive	41 (27-54)	11/183	0.603 (0.281 - 1.291)*	58 (35-82)	22/230	1.222 (0.750 - 1.992)	44 (35-66)	19/44	1.043 (0.623 - 1.745)*
T6 to T12, Multivariable	Changes to inactive	40 (26-54)	11/177	0.686 (0.312 - 1.510)	59 (35-83)	22/227	1.274 (0.777 - 2.088)	44 (35-66)	19/43	1.524 (0.850 - 2.732)

T6 to T12, Mult. (≥6 mo FU)	Changes to inactive	42 (30-55)	11/161	0.466 (0.187 - 1.164)*	63 (41-86)	18/209	1.182 (0.713 - 1.960)	44 (35-66)	19/43	1.249 (0.646 - 2.412)*
T6 to T12, Mult.+sociodemographics	Changes to inactive	40 (26-54)	11/176	0.678 (0.296 - 1.554)	59 (35-83)	22/225	1.227 (0.740 - 2.036)	44 (35-66)	19/43	1.449 (0.787 - 2.668)
T6 to T12, Mult. + PF	Changes to inactive	40 (26-54)	11/177	0.801 (0.360 - 1.779)*	58 (35-82)	21/224	1.283 (0.780 - 2.108)	44 (35-64)	19/42	1.542 (0.860 - 2.765)
T6 to T12, Unadjusted	Changes to active	38 (25-51)	9/170	0.604 (0.297 - 1.229)*	48 (28-68)	27/145	**0.504 (0.299 - 0.848)**	31 (20-44)	26/34	**0.366 (0.208 - 0.645)***
T6 to T12, Multivariable	Changes to active	39 (25-53)	9/161	0.718 (0.341 - 1.511)	48 (28-68)	27/144	**0.532 (0.314 - 0.900)**	31 (20-45)	24/32	**0.532 (0.287 - 0.987)**
T6 to T12, Mult. (≥6 mo FU)	Changes to active	41 (29-55)	6/142	0.717 (0.341 - 1.510)	50 (33-70)	25/132	**0.425 (0.242 - 0.744)***	37 (28-47)	18/26	0.554 (0.291 - 1.056)
T6 to T12, Mult.+sociodemographics	Changes to active	39 (25-53)	8/160	0.814 (0.383 - 1.730)*	48 (28-68)	26/143	**0.527 (0.310 - 0.898)**	31 (20-45)	24/32	**0.518 (0.276 - 0.975)**
T6 to T12, Mult. + PF	Changes to active	39 (25-53)	9/161	0.946 (0.441 - 2.031)*	48 (28-68)	27/143	**0.514 (0.301 - 0.877)**	31 (20-45)	24/32	0.550 (0.297 - 1.021)
T6 to T12, Unadjusted	Remains active	35 (24-52)	27/1132	**0.285 (0.166 - 0.491)**	46 (28-67)	86/924	**0.629 (0.432 - 0.916)**	36 (27-50)	57/96	0.675 (0.439 - 1.037)*
T6 to T12, Multivariable	Remains active	35 (24-52)	26/1096	**0.316 (0.175 - 0.571)**	46 (29-68)	84/908	**0.630 (0.429 - 0.927)**	36 (26-51)	56/94	0.899 (0.552 - 1.465)
T6 to T12, Mult. (≥6 mo FU)	Remains active	39 (28-55)	24/970	**0.293 (0.160 - 0.535)**	50 (33-69)	75/835	**0.566 (0.382 - 0.838)***	38 (29-51)	52/90	0.921 (0.543 - 1.560)
T6 to T12, Mult.+sociodemographics	Remains active	35 (24-52)	26/1091	**0.372 (0.202 - 0.684)***	46 (29-68)	82/900	**0.605 (0.405 - 0.902)**	36 (26-51)	56/94	0.935 (0.562 - 1.555)
T6 to T12, Mult. + PF	Remains active	35 (24-52)	26/1095	**0.449 (0.237 - 0.850)***	46 (29-68)	84/908	**0.639 (0.431 - 0.947)**	36 (26-51)	56/94	0.923 (0.565 - 1.509)
T6 to T12, Unadjusted	Continuous	37 (24-53)	72/1764	0.955 (0.724 - 1.317)	48 (30-71)	175/1542	0.832 (0.660 - 1.000)	36 (26-51)	135/219	**0.832 (0.724 - 0.955)**
T6 to T12, Multivariable	Continuous	37 (24-53)	67/1689	1.000 (0.724 - 1.378)	49 (30-71)	172/1512	0.832 (0.660 - 1.000)	36 (27-52)	130/212	**0.794 (0.660 - 1.000)**
T6 to T12, Mult. (≥6 mo FU)	Continuous	39 (28-55)	62/1501	1.047 (0.758 - 1.443)	52 (34-73)	157/1400	**0.794 (0.630 - 1.000)**	38 (29-54)	116/198	0.871 (0.691 - 1.096)
T6 to T12, Mult.+sociodemographics	Continuous	37 (24-53)	65/1679	1.000 (0.758 - 1.378)	49 (30-71)	168/1499	0.794 (0.660 - 1.000)	36 (27-52)	130/212	0.871 (0.724 - 1.047)
T6 to T12, Mult. + PF	Continuous	37 (24-53)	67/1688	1.000 (0.724 - 1.378)	49 (30-71)	171/1506	0.832 (0.660 - 1.000)	36 (26-52)	130/211	**0.794 (0.660 - 1.000)**
T12 to T24, Unadjusted	Remains inactive	46 (34-59)	13/168	Ref.	56 (38-78)	27/144	Ref.	47 (33-62)	15/21	Ref.
T12 to T24, Multivariable	Remains inactive	48 (35-60)	13/155	Ref.	58 (39-78)	26/137	Ref.	47 (35-64)	14/20	Ref.
T12 to T24, Mult. (≥6 mo FU)	Remains inactive	51 (38-62)	12/133	Ref.	61 (44-80)	24/123	Ref.	50 (36-66)	12/18	Ref.
T12 to T24, Mult.+sociodemographics	Remains inactive	48 (35-60)	12/153	Ref.	58 (39-78)	26/137	Ref.	47 (35-64)	14/20	Ref.
T12 to T24, Mult. + PF	Remains inactive	48 (35-60)	13/154	Ref.	57 (39-77)	25/134	Ref.	47 (35-64)	14/20	Ref.
T12 to T24, Unadjusted	Changes to inactive	43 (35-59)	4/115	NA	55 (39-76)	14/119	0.604 (0.316 - 1.152)	45 (35-71)	6/8	NA
T12 to T24, Multivariable	Changes to inactive	44 (35-58)	4/112	NA	55 (39-76)	14/117	0.616 (0.320 - 1.186)	53 (36-79)	5/7	NA
T12 to T24, Mult. (≥6 mo FU)	Changes to inactive	50 (39-61)	4/97	NA	57 (40-86)	14/107	0.644 (0.333 - 1.246)	58 (41-87)	4/6	NA
T12 to T24, Mult.+sociodemographics	Changes to inactive	44 (35-59)	4/111	NA	55 (39-76)	14/117	0.615 (0.319 - 1.187)	53 (36-79)	5/7	NA
T12 to T24, Mult. + PF	Changes to inactive	44 (35-58)	4/112	NA	55 (39-76)	14/117	0.693 (0.355 - 1.352)*	53 (36-79)	5/7	NA
T12 to T24, Unadjusted	Changes to active	41 (33-59)	3/113	NA	56 (38-81)	14/124	0.615 (0.322 - 1.172)	48 (42-62)	7/13	NA
T12 to T24, Multivariable	Changes to active	41 (33-59)	3/112	NA	56 (38-81)	14/121	0.626 (0.326 - 1.205)	48 (42-62)	7/13	NA
T12 to T24, Mult. (≥6 mo FU)	Changes to active	49 (38-62)	3/91	NA	60 (44-84)	11/110	0.653 (0.338 - 1.261)	48 (42-62)	7/13	NA
T12 to T24, Mult.+sociodemographics	Changes to active	41 (33-59)	3/111	NA	56 (38-81)	14/121	0.647 (0.335 - 1.248)	48 (42-62)	7/13	NA
T12 to T24, Mult. + PF	Changes to active	41 (33-59)	3/112	NA	56 (38-81)	14/121	0.696 (0.359 - 1.351)*	48 (42-62)	7/13	NA
T12 to T24, Unadjusted	Remains active	43 (34-59)	15/748	NA	57 (38-77)	54/723	**0.403 (0.254 - 0.641)**	49 (38-63)	28/66	NA
T12 to T24, Multivariable	Remains active	43 (34-59)	15/725	NA	57 (38-77)	53/709	**0.395 (0.246 - 0.636)**	49 (38-63)	28/66	NA
T12 to T24, Mult. (≥6 mo FU)	Remains active	50 (38-61)	12/612	NA	61 (45-80)	51/627	**0.413 (0.255 - 0.668)**	49 (39-63)	27/64	NA

T12 to T24, Mult.+sociodemographics	Remains active	43 (34-59)	15/719	NA	57 (38-77)	53/704	**0.394 (0.242 - 0.640)**	49 (38-63)	28/66	NA
T12 to T24, Mult. + PF	Remains active	43 (34-59)	15/725	NA	57 (38-77)	52/706	**0.436 (0.264 - 0.721)***	48 (37-63)	28/65	NA
T12 to T24, Unadjusted	Continuous	43 (34-59)	35/1144	NA	56 (38-78)	109/1110	0.955 (0.724 - 1.258)	48 (36-63)	56/108	NA
T12 to T24, Multivariable	Continuous	44 (34-59)	35/1104	NA	57 (38-78)	107/1084	0.955 (0.724 - 1.258)	48 (36-63)	54/106	NA
T12 to T24, Mult. (≥6 mo FU)	Continuous	50 (38-61)	31/933	NA	60 (44-81)	100/967	0.955 (0.724 - 1.258)	49 (38-64)	50/101	NA
T12 to T24, Mult.+sociodemographics	Continuous	44 (34-59)	34/1094	NA	57 (38-78)	107/1079	0.955 (0.724 - 1.258)	48 (36-63)	54/106	NA
T12 to T24, Mult. + PF	Continuous	44 (34-59)	35/1103	NA	57 (38-78)	105/1078	0.912 (0.691 - 1.258)	48 (36-63)	54/105	NA

Effect estimates for continuous activity are shown per 1 SD increase (46 MET-hrs/wk). PA categorization was based on cut-offs from tertiles created in an age- and sex-matched sample of the general population. Low (reference group): tertile 1, moderate: tertile 2, high: tertile 3. Remains inactive (reference group): low at both timepoints; change to inactive: moderate/high at first- and low at second timepoint; change to active: low at first- and moderate/high at second timepoint; remains active: moderate/high at both timepoints.
Unadjusted models included only PA as exposure. Mult. (≥6 mo FU): restriction to patients with ≥6 months FU after last used PA questionnaire. Mult. (expanded): further adjustment for smoking status (current, prior, former), education (low, moderate, high), marital status (single, married/in-law). Mult. + PF2: further adjustment for functional status using the revised physical functioning (PF2)_subscale of the EORTC QLQ-C30 at the time of first of the 2 subsequent PA assessments (dichotomized at >66.7 vs. ≤66.7).
*Estimates that changed >10% compared to the main multivariable model.
Abbreviations: CRC, colorectal cancer; FU, FU time in months; IQR, interquartile range; HR, hazard ratio; CI, confidence interval; Ref., reference; PA, physical activity; MET-hrs/wk, metabolic equivalent of task-hours per week; EORTC, European Organisation for Research and Treatment of Cancer; QLQ-C30, Quality of Life Questionnaire-Core 30; PF2, PF subscale (second version).

Fig S1 – Recreational physical activity descriptives over time

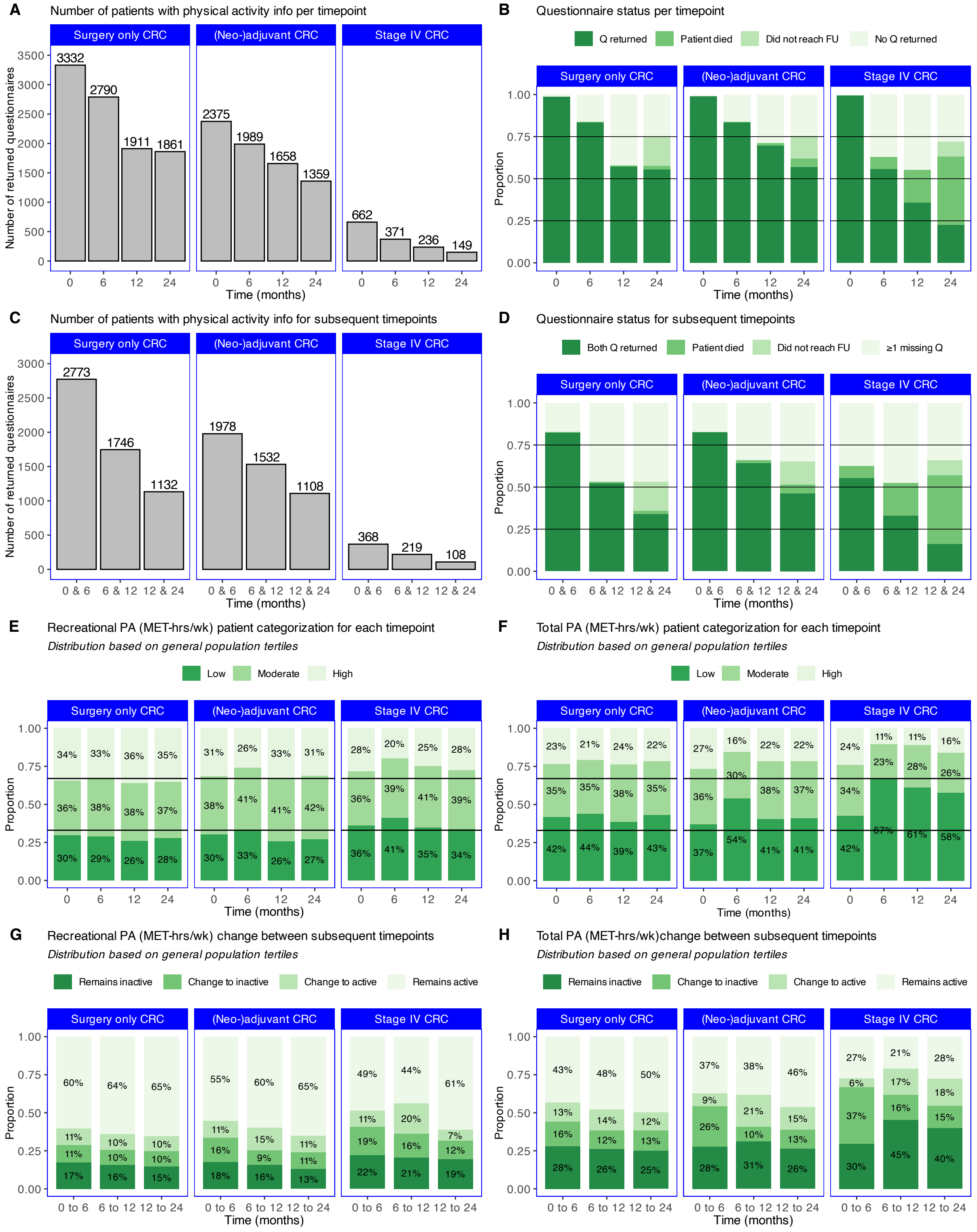

(A) Number of patients with returned PA questionnaires for each timepoint. (B) Proportions of returned questionnaires and reasons for missingness for each timepoint. (C) Number of patients with returned questionnaires at two subsequent timepoints. (D) Proportions of two subsequently returned questionnaires and reasons for missingness. (E, F) PA categorization was based on cut-offs from tertiles created in an age- and sex-matched sample of the general population. Low: tertile 1, moderate: tertile 2, high: tertile 3. (G, H) PA categorization between subsequent timepoints was based on the general population tertiles. Remains inactive: tertile 1 at both timepoints; change to inactive: tertile 2/3 at first- and tertile 1 at second timepoint; change to active: tertile 2/3 at first- and tertile 1 at second timepoint; remains active: tertile 2/3 at both timepoints.

Abbreviations: CRC, colorectal cancer; PA, physical activity; MET-hrs/wk, metabolic equivalent of task-hours per week; Q, questionnaire; FU, FU.

Fig S2 – Associations from Cox proportional hazard models for timepoint associations of total physical activity (MET-hours per week), moderate and vigorous physical activity (MVPA, MET-hours per week) and adherence to PA guidelines with overall survival

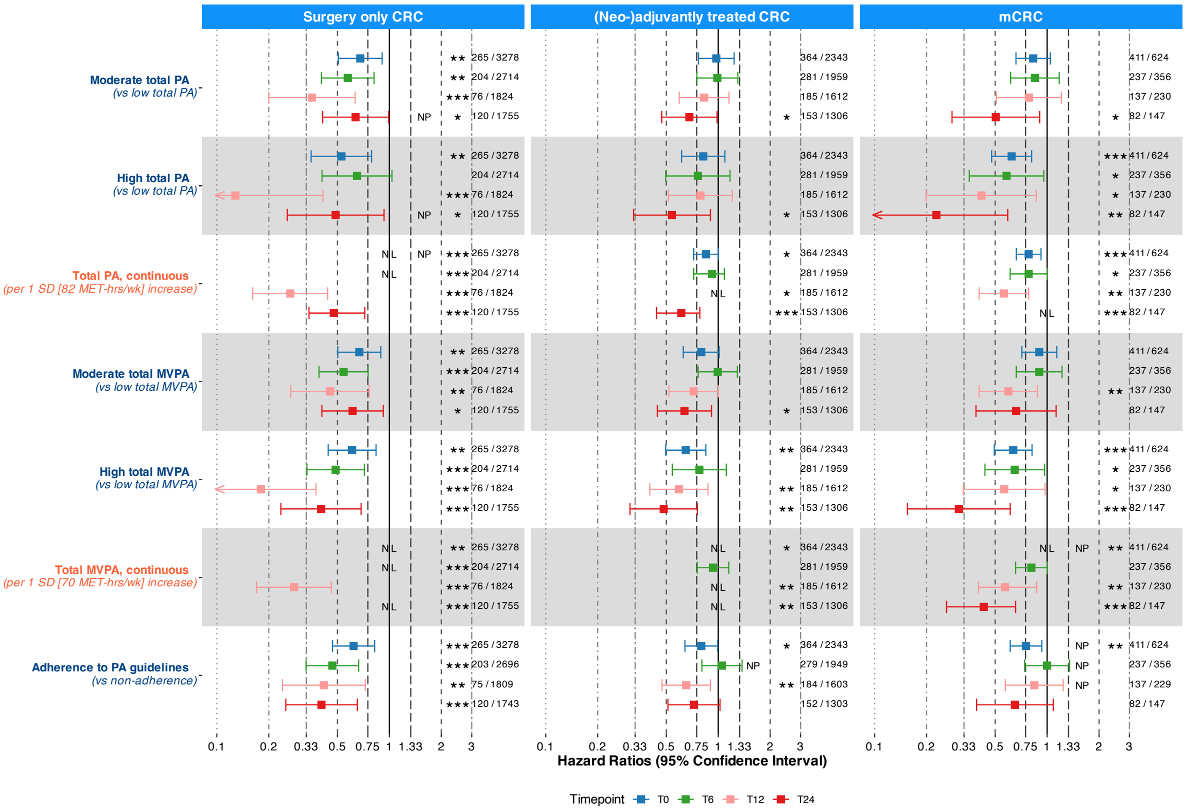

PA categorization was based on cut-offs from tertiles created in an age- and sex-matched sample of the general population. Low: tertile 1, moderate: tertile 2, high: tertile 3.

Numbers depict deaths / total patients in analysis. Stars indicate significant associations (*: p = 0.01 to <0.05; **: p = 0.001 to <0.01; *** p <0.001). NL indicates a nonlinear association, NP that the Proportional hazards assumption is violated. Fixed covariates: age (continuous), sex (male, female), primary tumor site (colon, rectum), cohort (PLCRC, COLON). Covariates at time of PA measurement: BMI (18.5-25, other), stoma (yes, no). Additional covariates in stage IV analyses: number of metastases (1, >1), liver-only metastasis (yes, no), surgery of primary tumor (yes, no), metastasectomy (yes, no), additional treatment during first disease episode (none, chemotherapy, radiotherapy, both).

Abbreviations: CRC, colorectal cancer; PA, physical activity, MVPA: moderate and vigorous physical activity; HR, hazard ratio; CI, confidence interval; MET-hrs/wk, metabolic equivalent of task-hours per week.

Fig S3 – Restricted cubic splines from Cox proportional hazard models for timepoint associations of total physical activity (MET-hours per week) with overall survival

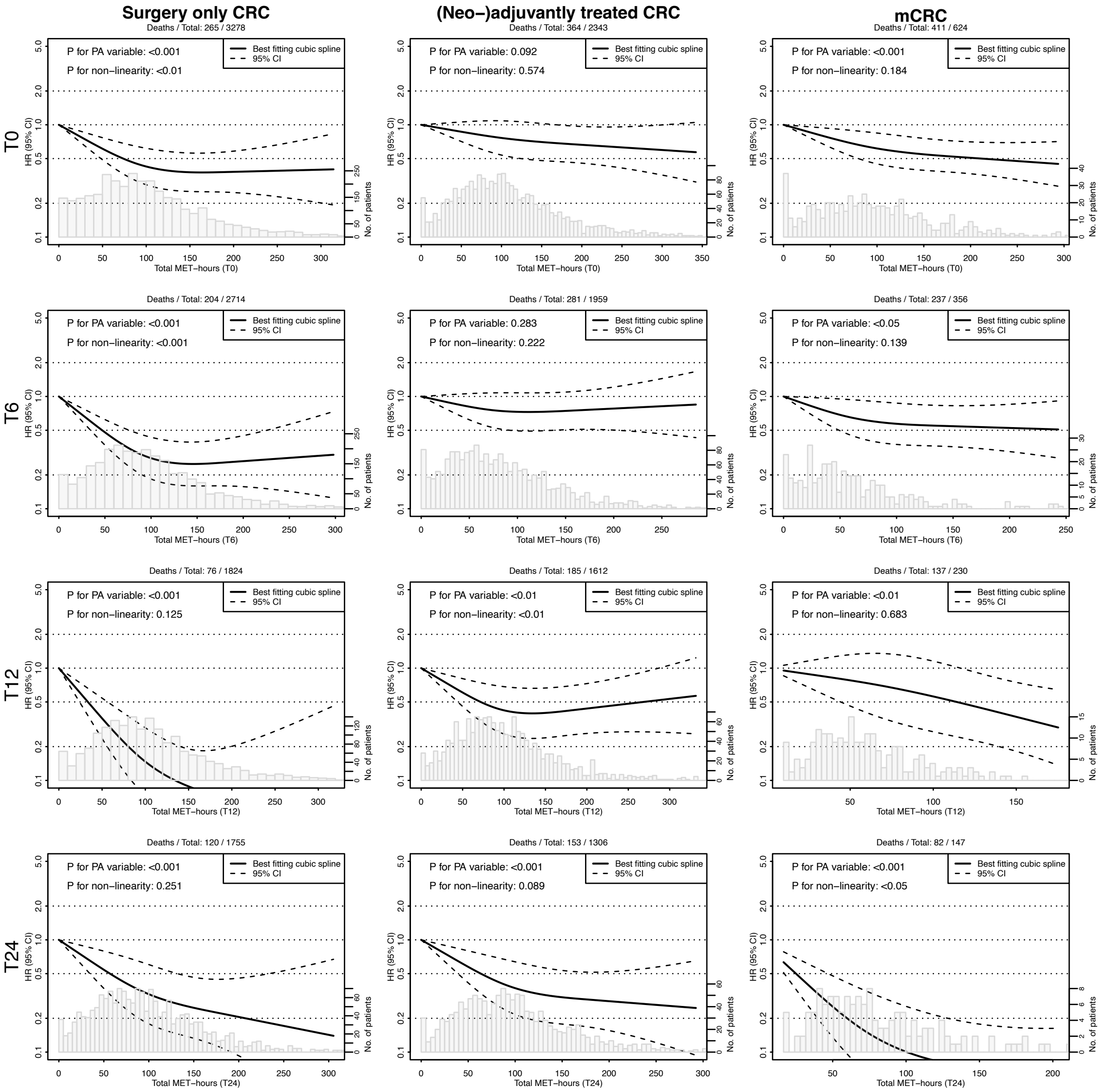

Plot curves were estimated using restricted cubic spline regressions with three knots placed at fixed percentiles (10%, 50%, and 90%) of the physical activity variable, and 0 MET-hours was chosen as reference category. Note that the number of deaths is below the number of variables divided by 10 for T24 for mCRC analyses, possibly resulting in unstable estimates. Abbreviations: CRC, colorectal cancer; PA, physical activity, HR, hazard ratio; CI, confidence interval; MET-hrs/wk, metabolic equivalent of task-hours per week.

Fig S4 – Restricted cubic splines from Cox proportional hazard models for timepoint associations of total moderate and vigorous physical activity (MVPA, MET-hours per week) with overall survival

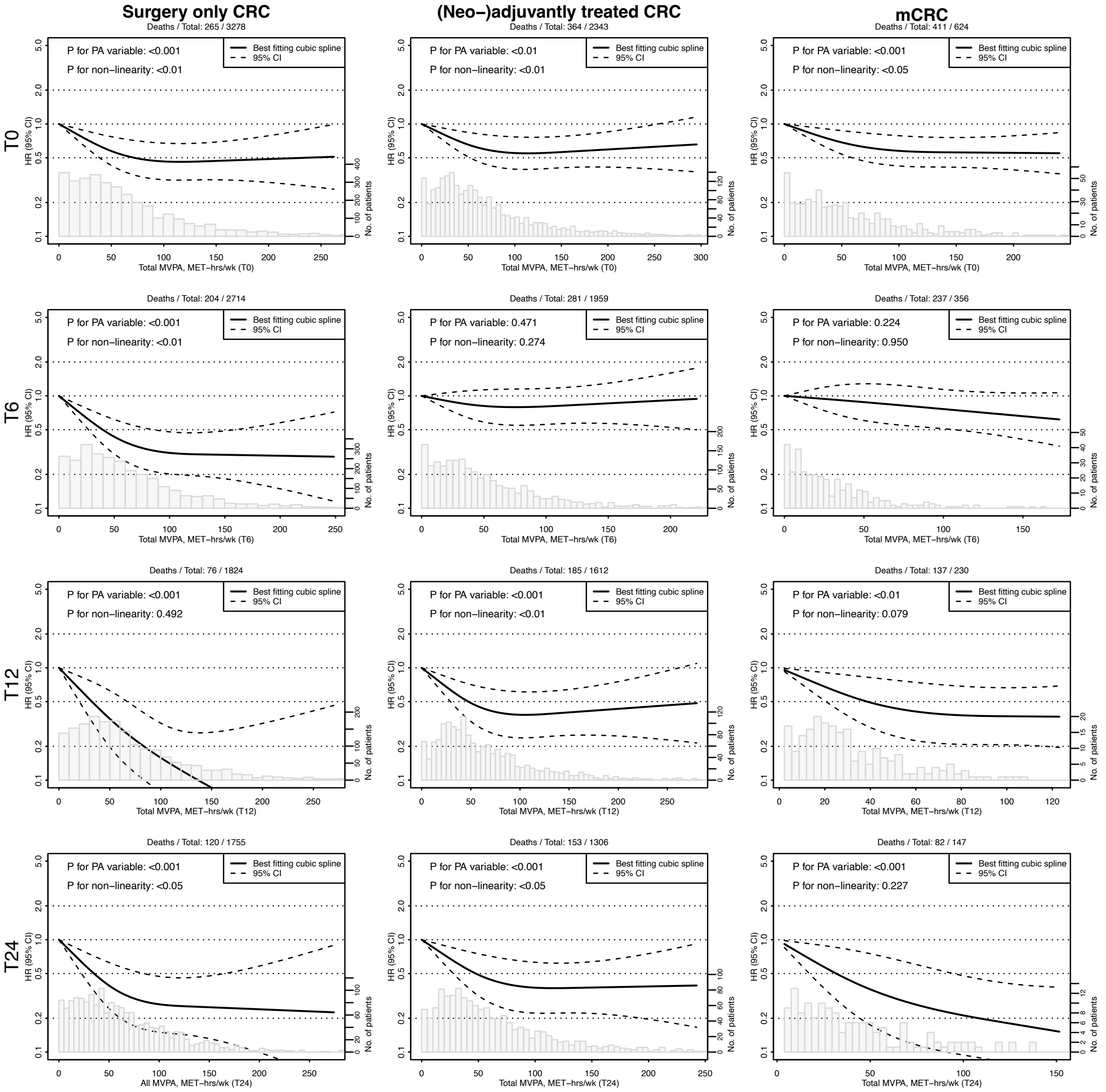

Plot curves were estimated using restricted cubic spline regressions with three knots placed at fixed percentiles (10%, 50%, and 90%) of the physical activity variable, and 0 MET-hours was chosen as reference category. Note that the number of deaths is below the number of variables divided by 10 for T24 for mCRC analyses, possibly resulting in unstable estimates. Abbreviations: CRC, colorectal cancer; PA, physical activity, HR, hazard ratio; CI, confidence interval; MVPA, moderate and vigorous physical activity; MET-hrs/wk, metabolic equivalent of task-hours per week.

Fig S5 – Associations from Cox proportional hazard models for change between subsequent timepoint associations of total physical activity (MET-hours per week), total moderate and vigorous physical activity (MVPA, MET-hours per week), and adherence to physical activity guidelines with overall survival

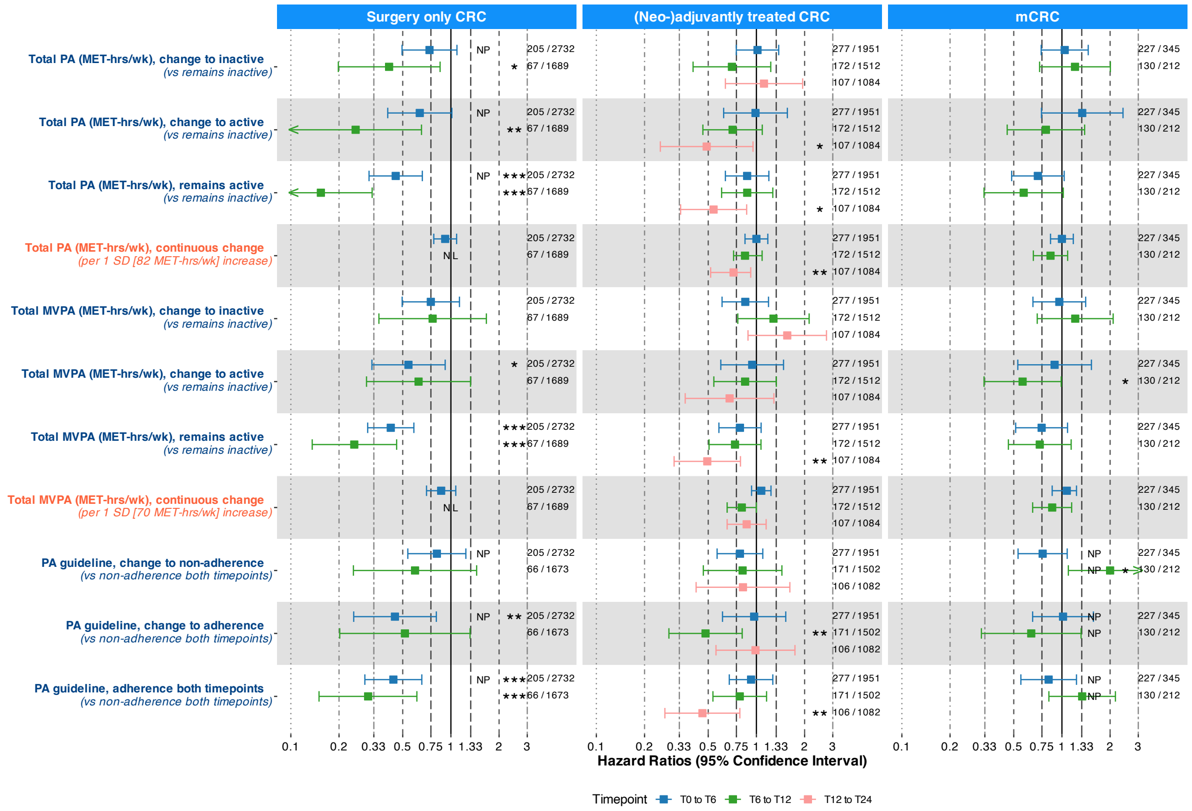

PA categorization was based on cut-offs from tertiles created in an age- and sex-matched sample of the general population. Low: tertile 1, moderate: tertile 2, high: tertile 3. Remains inactive: tertile 1 at both timepoints; change to inactive: tertile 2/3 at first- and tertile 1 at second timepoint; change to active: tertile 2/3 at first- and tertile 1 at second timepoint; remains active: tertile 2/3 at both timepoints. T12 to T24 change analyses were removed for surgery only CRC and mCRC due to limited events.

Numbers depict deaths / total patients in analysis. Stars indicate significant associations (*: $p = 0.01$ to <0.05; **: $p = 0.001$ to <0.01; *** $p <0.001$). NL indicates a nonlinear association, NP that the Proportional hazards assumption is violated. Fixed covariates: age (continuous), sex (male, female), primary tumor site (colon, rectum), cohort (PLCRC, COLON). Covariates at time of PA measurement: BMI (18.5-25, other), stoma (yes, no). Additional covariates in stage IV analyses: number of metastases (1, >1), liver-only metastasis (yes, no), surgery of primary tumor (yes, no), metastasectomy (yes, no), additional treatment during first disease episode (none, chemotherapy, radiotherapy, both).

Abbreviations: CRC, colorectal cancer; PA, physical activity, MVPA: moderate and vigorous physical activity; HR, hazard ratio; CI, confidence interval; MET-hrs/wk, metabolic equivalent of task-hours per week.

Data supplement, Fig 6 – Restricted cubic splines from Cox proportional hazard models for change between subsequent timepoints associations of total physical activity (MET-hours per week) with overall survival

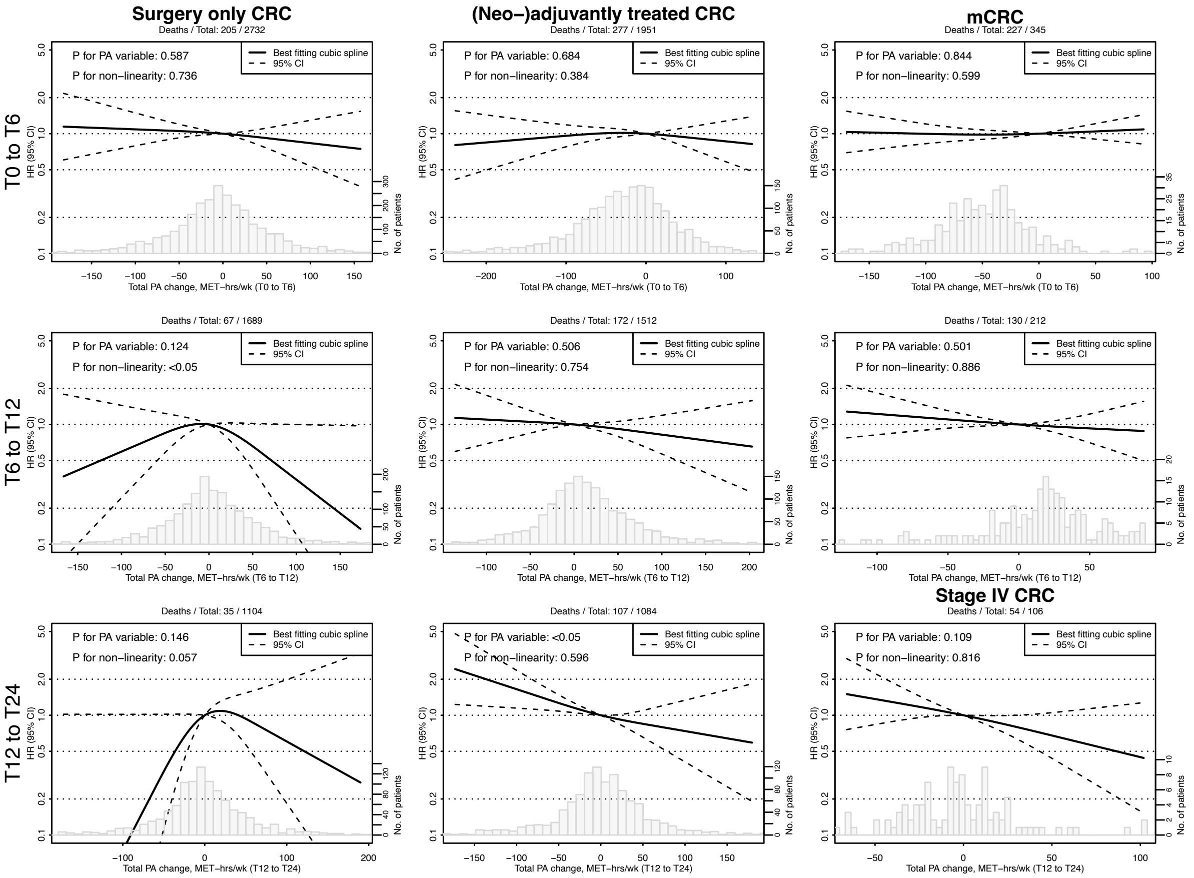

Plot curves were estimated using restricted cubic spline regressions with three knots placed at fixed percentiles (10%, 50%, and 90%) of the physical activity variable, and 0 MET-hours was chosen as reference category. Note that the number of deaths is below the number of variables divided by 10 for T12 to T24 for both surgery only CRC and mCRC analyses, possibly resulting in unstable estimates.

Abbreviations: CRC, colorectal cancer; PA, physical activity, HR, hazard ratio; CI, confidence interval; MVPA, moderate and vigorous physical activity; MET-hrs/wk, metabolic equivalent of task-hours per week.

Fig S7 – Restricted cubic splines from Cox proportional hazard models for change between subsequent timepoints associations of total moderate and vigorous physical activity (MVPA, MET-hours per week)

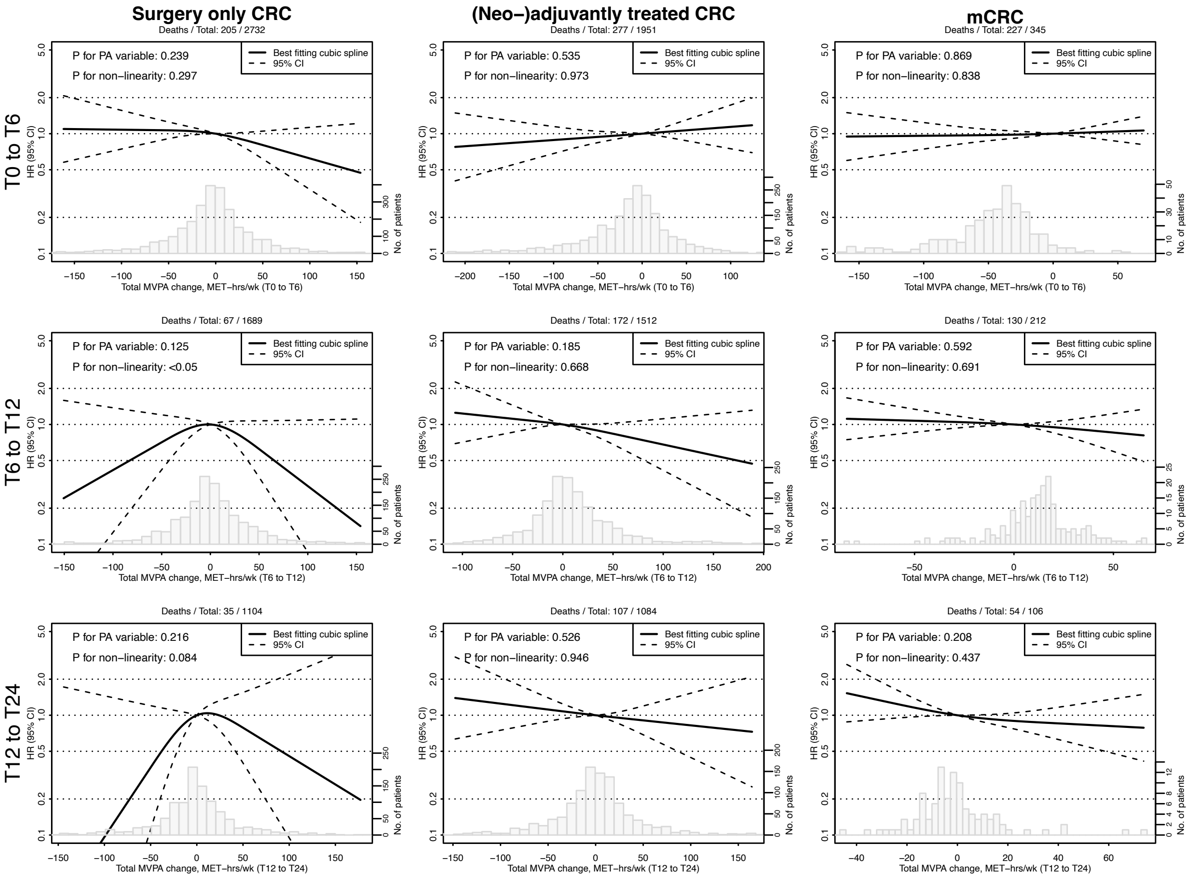

Plot curves were estimated using restricted cubic spline regressions with three knots placed at fixed percentiles (10%, 50%, and 90%) of the physical activity variable, and 0 MET-hours was chosen as reference category. Note that the number of deaths is below the number of variables divided by 10 for T12 to T24 for both surgery only CRC and mRC analyses, possibly resulting in unstable estimates.

Abbreviations: CRC, colorectal cancer; PA, physical activity, HR, hazard ratio; CI, confidence interval; MVPA, moderate and vigorous physical activity; MET-hrs/wk, metabolic equivalent of task-hours per week.

Fig S8 – Associations from Cox proportional hazard models for timepoint associations of recreational physical activity (MET-hours per week) with overall survival for stage I to III and stage I to IV colorectal cancer patients.

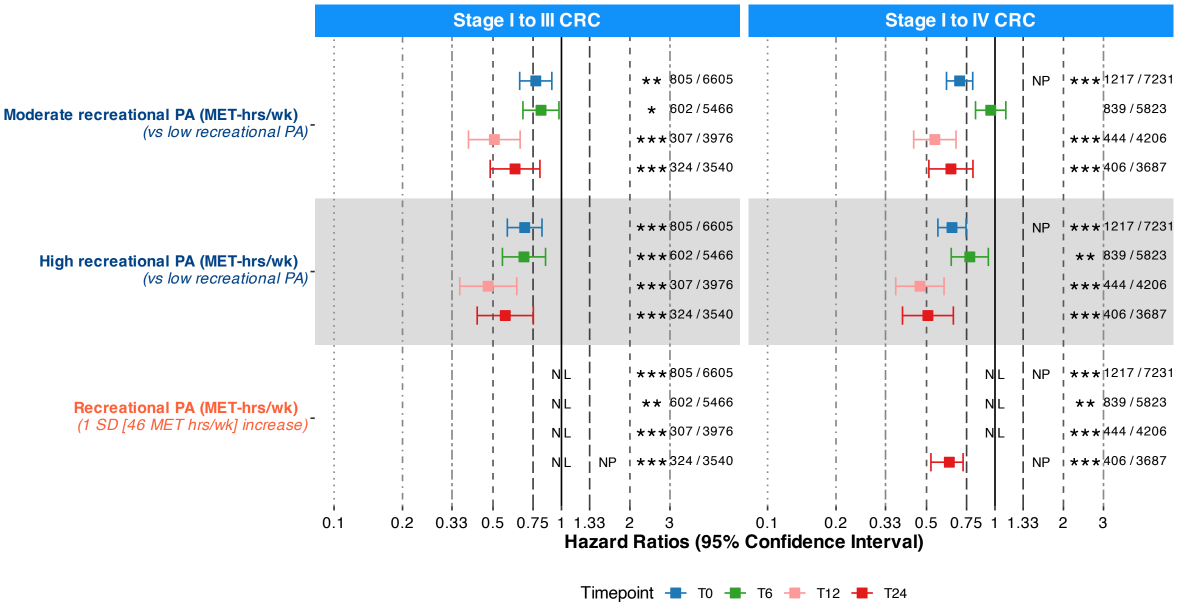

PA categorization was based on cut-offs from tertiles created in an age- and sex-matched sample of the general population. Low: tertile 1, moderate: tertile 2, high: tertile 3.
Numbers depict deaths / total patients in analysis. Stars indicate significant associations (*: p = 0.01 to <0.05; **: p = 0.001 to <0.01; *** p <0.001). NL indicates a nonlinear association, NP that the Proportional hazards assumption is violated. Fixed covariates: age (continuous), sex (male, female), primary tumor site (colon, rectum), tumor stage (I, II, III, IV), cohort (PLCRC, COLON). Covariates at time of PA measurement: BMI (18.5-25, other), stoma (yes, no).
Abbreviations: CRC, colorectal cancer; PA, physical activity, HR, hazard ratio; CI, confidence interval; MET-hrs/wk, metabolic equivalent of task-hours per week.

Fig S9 – Restricted cubic splines from Cox proportional hazard models for timepoint associations of recreational physical activity (MET-hours per week) with overall survival for stage I to III and stage I to IV CRC patients

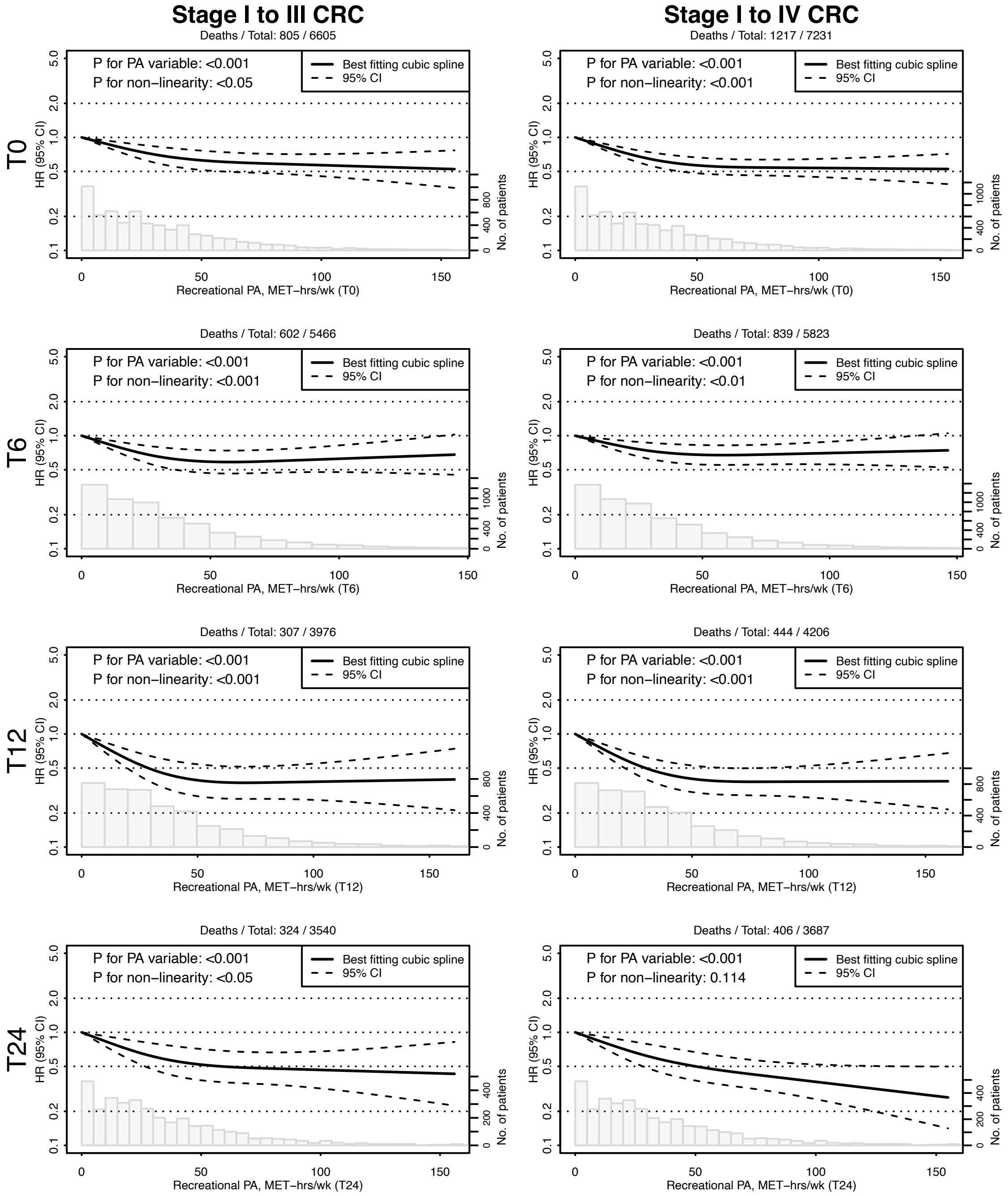

Plot curves were estimated using restricted cubic spline regressions with three knots placed at fixed percentiles (10%, 50%, and 90%) of the physical activity variable, and 0 hours was chosen as reference category.
Abbreviations: CRC, colorectal cancer; PA, physical activity, HR, hazard ratio; CI, confidence interval; MET-hrs/wk, metabolic equivalent of task-hours per week.

Fig S10 – Associations from Cox proportional hazard models for change between subsequent timepoints associations of recreational physical activity (MET-hours per week) with overall survival for stage I to III and stage I to IV CRC patients.

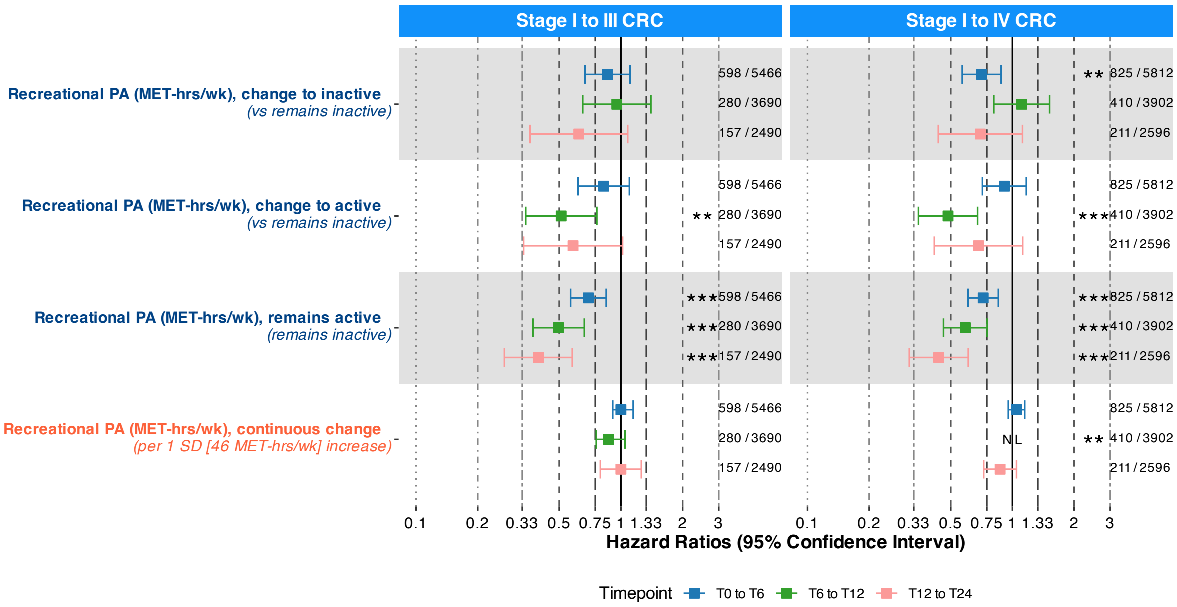

PA categorization was based on cut-offs from tertiles created in an age- and sex-matched sample of the general population. Low: tertile 1, moderate: tertile 2, high: tertile 3. Remains inactive: tertile 1 at both timepoints; change to inactive: tertile 2/3 at first- and tertile 1 at second timepoint; change to active: tertile 2/3 at first- and tertile 1 at second timepoint; remains active: tertile 2/3 at both timepoints.

Numbers depict deaths / total patients in analysis. Stars indicate significant associations (*: p = 0.01 to <0.05; **: p = 0.001 to <0.01; *** p <0.001). NL indicates a nonlinear association, NP that the Proportional hazards assumption is violated. Fixed covariates: age (continuous), sex (male, female), primary tumor site (colon, rectum), tumor stage (I, II, III, IV), cohort (PLCRC, COLON). Covariates at time of PA measurement: BMI (18.5-25, other), stoma (yes, no).

Abbreviations: CRC, colorectal cancer; PA, physical activity, HR, hazard ratio; CI, confidence interval; MET-hrs/wk, metabolic equivalent of task-hours per week.

Fig S11 – Restricted cubic splines from Cox proportional hazard models for change between subsequent timepoints associations of recreational physical activity (MET-hours per week) with overall survival for stage I to III and stage I to IV CRC patients

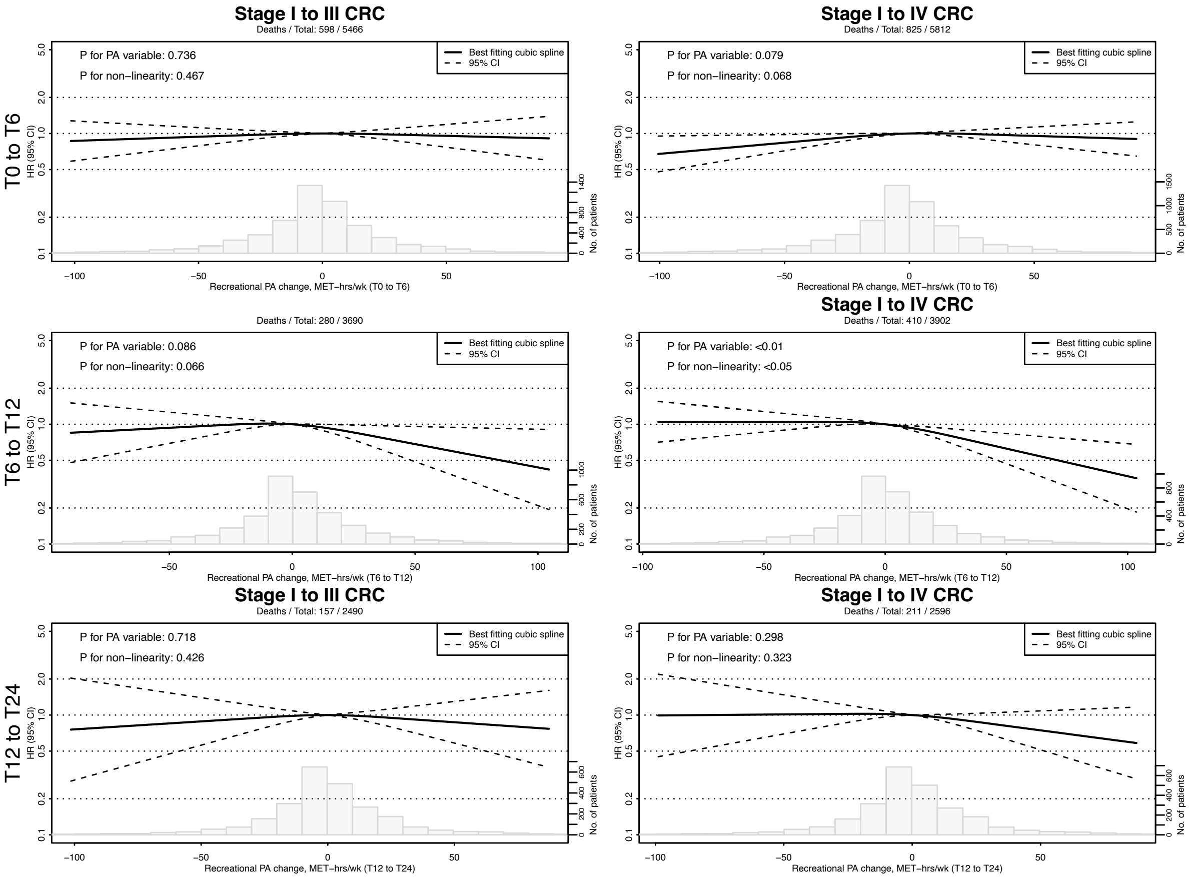

Plot curves were estimated using restricted cubic spline regressions with three knots placed at fixed percentiles (10%, 50%, and 90%) of the physical activity variable, and 0 hours was chosen as reference category.
Abbreviations: CRC, colorectal cancer; PA, physical activity, HR, hazard ratio; CI, confidence interval; MET-hrs/wk, metabolic equivalent of task-hours per week.

Fig S12 – Sensitivity analyses where missing PA values are revalued to either low or high for recreational physical activity (MET-hours/week)

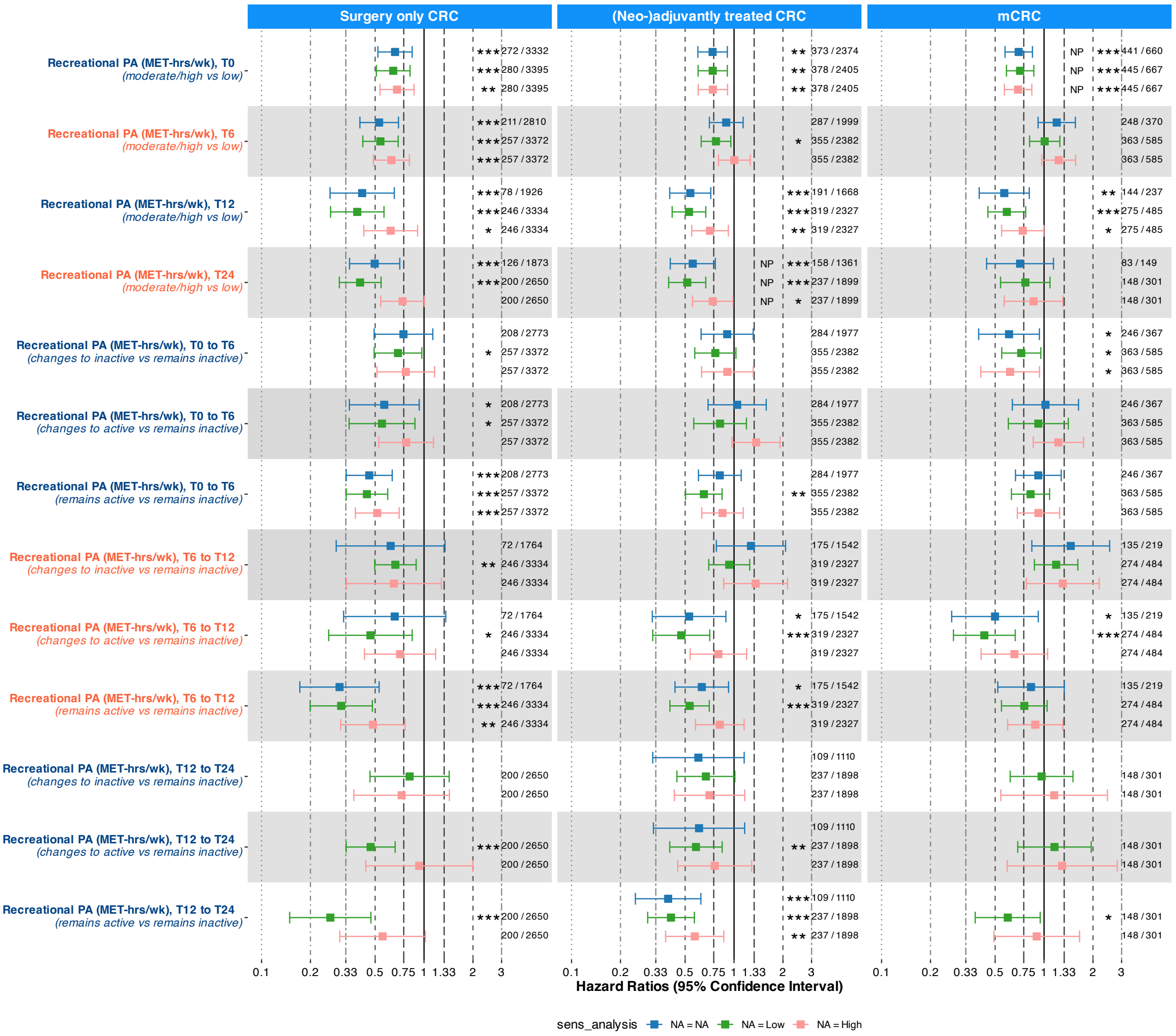

PA categorization was based on cut-offs from tertiles created in an age- and sex-matched sample of the general population. Low: tertile 1, moderate: tertile 2, high: tertile 3. Remains inactive: tertile 1 at both timepoints; change to inactive: tertile 2/3 at first- and tertile 1 at second timepoint; change to active: tertile 2/3 at first- and tertile 1 at second timepoint; remains active: tertile 2/3 at both timepoints.

Patients with FU time ≥3 months more than the (last) used questionnaire are either revalued to low or moderate/high physical activity. Original estimates for surgery only CRC and mCRC between T12 and T24 are not shown due to insufficient events. Numbers depict deaths / total patients in analysis. Stars indicate significant associations (*: p = 0.01 to <0.05; **: p = 0.001 to <0.01; *** p <0.001). NP indicates that the Proportional hazards assumption is violated. Fixed covariates: age (continuous), sex (male, female), primary tumor site (colon, rectum), cohort (PLCRC, COLON). Additional covariates in stage IV analyses: number of metastases (1, >1), liver-only metastasis (yes, no), surgery of primary tumor (yes, no), metastasectomy (yes, no), additional treatment during first disease episode (none, chemotherapy, radiotherapy, both). Covariates at time of PA measurement are removed from analyses: BMI (18.5-25, other), stoma (yes, no).

Abbreviations: CRC, colorectal cancer; PA, physical activity, HR, hazard ratio; CI, confidence interval; MET-hrs/wk, metabolic equivalent of task-hours per week; NA, missing.
